# Co-inoculation of *Bacillus subtilis* and *Bradyrhizobium liaoningense* increased soybean yield and improved soil bacterial community composition in coastal saline-alkali land

**DOI:** 10.3389/fpls.2025.1677763

**Published:** 2025-10-14

**Authors:** Caizhi He, Tongguo Gao, Xinxin Wang, Renqiang Chen, Huiyan Gao, Hongquan Liu

**Affiliations:** ^1^ College of Urban and Rural Construction Hebei Agricultural University, Baoding, China; ^2^ College of Life Sciences Hebei Agricultural University, Baoding, China; ^3^ Agricultural Technology Innovation Center in Mountainous Areas of Hebei Province, Baoding, China; ^4^ Agricultural Engineering Technology Research Center of National North Mountainous Area, Baoding, China; ^5^ Hebei Mountain Research Institute, Hebei Agricultural University, Baoding, China; ^6^ North China Water Saving Agriculture Key Laboratory of the Ministry of Agriculture and Rural Affairs, Baoding, China; ^7^ State Key Laboratory of North China Crop Improvement and Regulation, Baoding, China

**Keywords:** bacterial community, coastal saline-alkali land, enzyme activity, microbial inoculum co-inoculation, salt-tolerant soybean

## Abstract

Saline-alkali land is an important reserve cultivated land resource, and increasing soybean yield in it is significant for food security. As soybean is sensitive to saline-alkali stress, planting it in such soil often causes problems like blocked root development and yield decline. In this study, field experiments were carried out in coastal saline-alkali land in Huanghua City, Hebei Province. Jidou 12 with strong saline-alkali tolerance was selected, and four treatments (CK:conventional fertilization;T1:conventional fertilization + 75L/hm^2^ *Bacillus subtilis* 8–32 agent; T2:conventional fertilization + 75L/hm^2^ *Bradyrhizobium liaoningense* CCBAU05525;T3:conventional fertilization + 75L/hm^2^ *Bacillus subtilis* 8–32 agent and 75L/hm^2^ *Bradyrhizobium liaoningense* CCBAU05525). Through data analysis, the effects of co-inoculation of *Bacillus subtilis* and *Rhizobium* on soybean yield, soil properties, enzyme activity and bacterial community composition were studied. The results showed that T3 significantly increased soybean yield to 3182.67 kg/hm^2^, with yield, grains per pod and 100-grain weight increasing by 18.03%, 18.6% and 2.7% respectively compared with CK. The pH, electrical conductivity and total water-soluble salt content of rhizosphere soil decreased by 2.8%, 11.0% and 5.4%, while water and organic matter content increased by 5.6% and 11.6%. The activities of alkaline phosphatase, sucrase, urease and catalase increased by 14.9%, 22.4%, 15.1% and 5.2%. Co-inoculation increased the relative abundance of Sphingomonas. There was no significant difference in the Ace and Chao indices, indicating no significant difference in OTU number. The Shannon index of T1 was lower, meaning lower bacterial community species diversity. Co-inoculation improved plant stress resistance by enhancing the rhizosphere soil environment, regulating the microbial community structure and soil salinity, promoting soybean yield formation. It provides a theoretical basis for scientific fertilization and soil improvement in saline-alkali soybean planting.

## 1 Introduction

At present, more than 100 countries in the world are affected by soil salinization. China’s saline-alkali land area is about 7.67×10^6^hm^2^, which is the third largest saline-alkali land distribution area in the world (Wu et al., 2023), mainly distributed in the northwest, north, northeast and coastal areas. Among them, the total area of saline-alkali cultivated land in Hebei is 7.8×10^5^hm^2^, accounting for 10.4% of the total area of cultivated land. Huanghua City is located in the eastern part of Cangzhou City, Hebei Province, adjacent to the Bohai Sea. It is a typical area of the Bohai Sea saline-alkali belt. Soil salinization is a worldwide problem affecting agricultural production (Peng et al., 2025). The development and utilization of saline-alkali land is of great significance to food security, sustainable agricultural development and farmers’ income increase (Li Y, et al., 2024).

Soybean is an important food crop and oil crop. China is the world’s largest soybean importer and consumer (Zhang and Shi, 2024).In 2023, China ‘s soybean consumption was about 115 million tons, accounting for 1/3 of the total global consumption (Hu and Si, 2025).In the same year, China ‘s soybean import volume was 99.41 million tons, while China ‘s total soybean output was only 20.84 million tons (Li and He, 2024).In China, where cultivated land resources are limited, using saline-alkali land to grow soybeans is an effective way to increase soybean production. However, under salt stress, soybean showed poor root development, decreased nitrogen and phosphorus absorption efficiency, and weakened symbiotic nitrogen fixation ability (Ma et al., 2025), which directly affected soybean yield.

Using plant growth promoting rhizobacteria (PGPR) to improve the salt and alkali tolerance of crops has become an effective way to increase yield (Ali et al., 2025; Liu et al., 2016; Zhuang et al., 2023). Several PGPR strains have been shown to efficiently produce plant growth promoting substances under salt stress, and to enhance plant stress resistance and growth performance by regulating physiological and biochemical signals within plants (Nader et al., 2024).Co-inoculation of plant growth promoting bacteria and *Rhizobium* was also confirmed to significantly promote soybean growth, nodulation and nitrogen uptake efficiency. The nodule dry weight and total plant nitrogen content were higher than those of single inoculation (Cheng et al., 2025; Fonseca de Souza et al., 2025). However, there are few studies on the effects of co-inoculation of plant growth-promoting rhizobacteria and *Rhizobium* on soybean growth and rhizosphere soil microbial structure under saline-alkali conditions.

In the previous study, a strain of *Bacillus subtilis* 8–32 with high yield of indole acetic acid (IAA) was screened. In this study, typical coastal saline-alkali land in China was selected, and Jidou 12 with strong saline-alkali tolerance was used as the test soybean variety. The effects of *Bacillus subtilis* 8–32 and *Bradyrhizobium liaoningense* CCBAU05525 co-inoculation on soybean yield, soil physical and chemical properties, soil enzyme activity and soil bacterial community composition were studied. The high-yield planting mode of soybean in saline-alkali land was proposed, which provided theoretical basis and technical support for food security and comprehensive development and utilization of saline-alkali land.

## 2 Materials and methods

### 2.1 Experiment field

**Figure 1:**
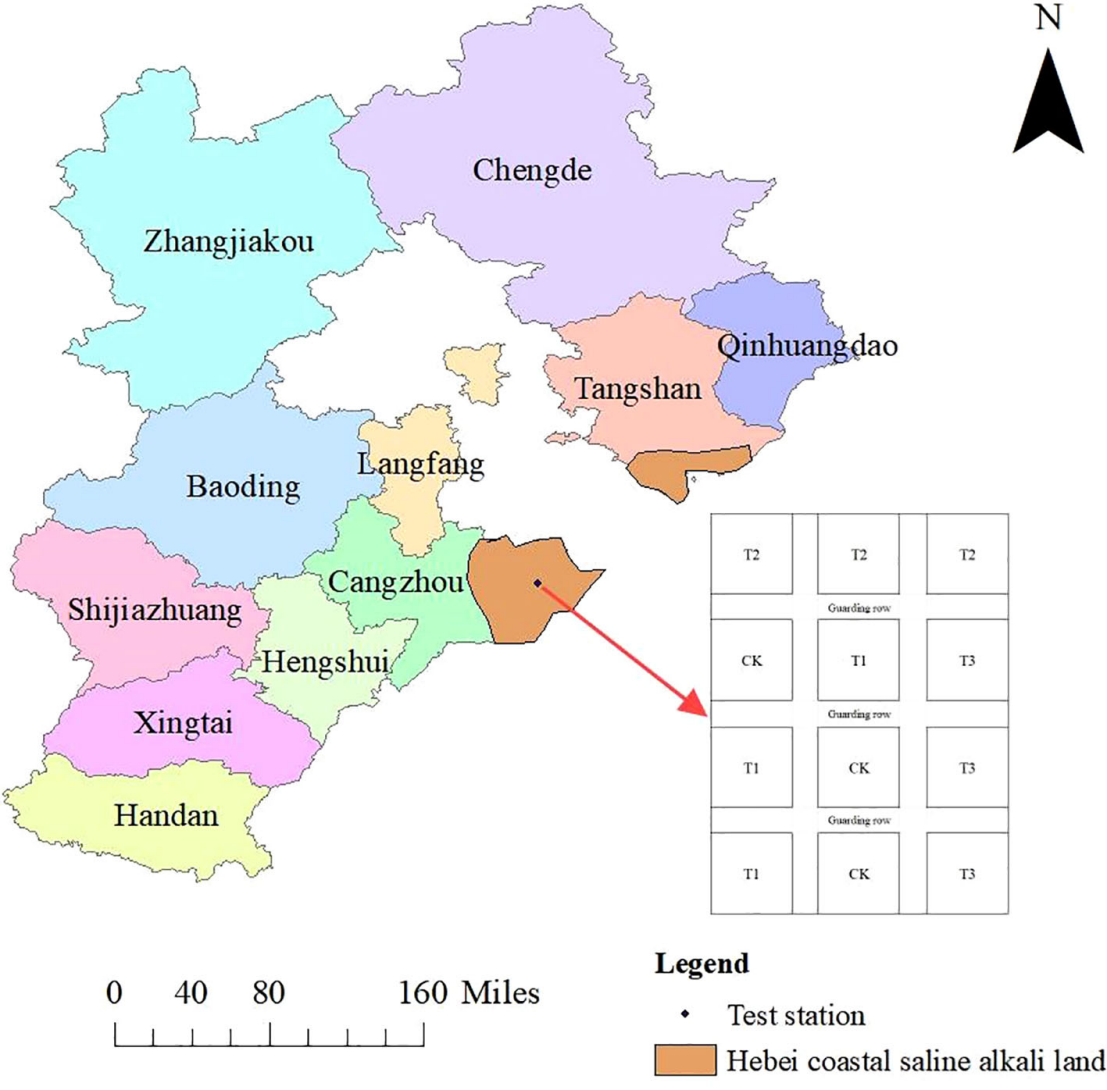
Overview of the experimental site.

**Figure 2:**
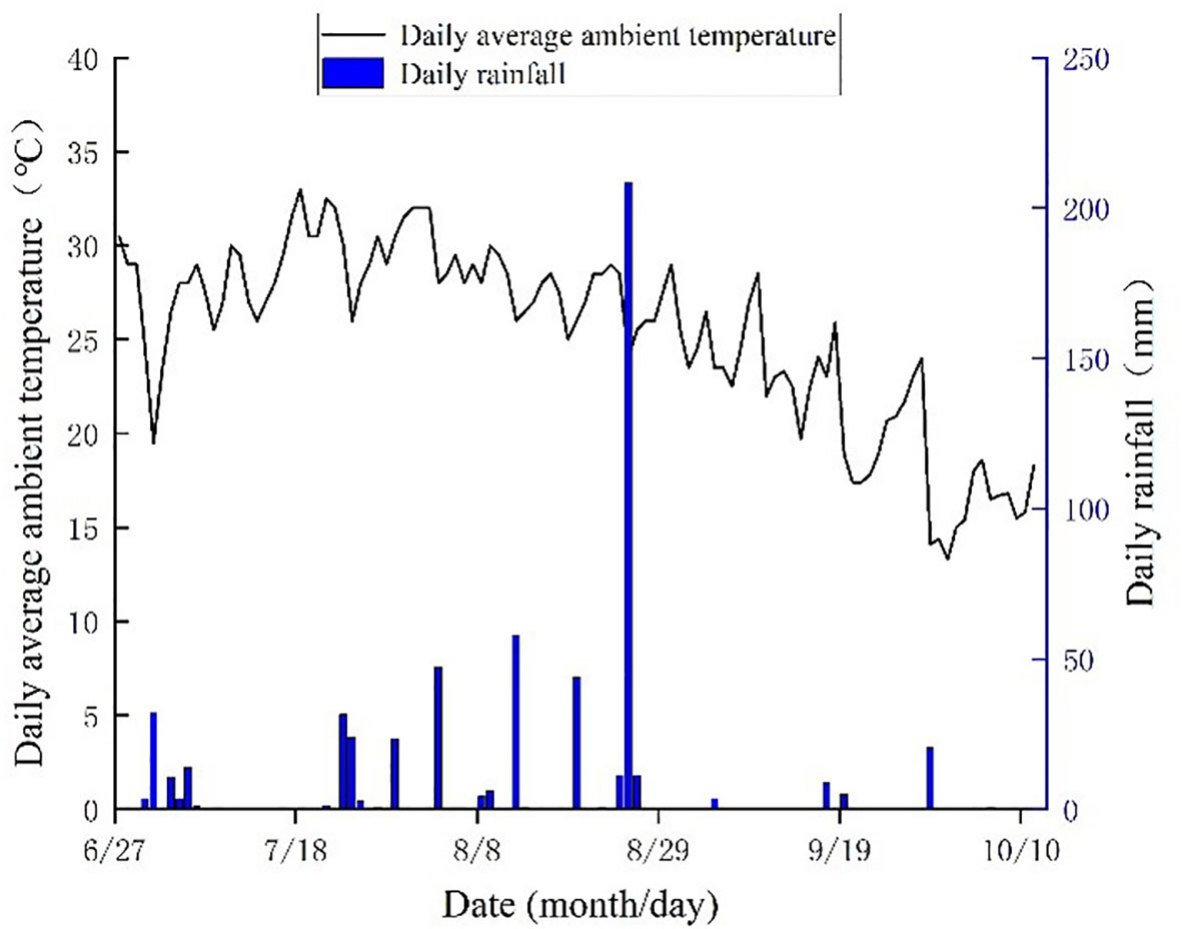
Variation of main meteorological elements during soybean growth period in 2024.

The field experiment was carried out from June to October 2024 in Youhe Planting Cooperative, which is located at Lizizha Village, Changguo Town, Huanghua City, Hebei Province. The geographical location is 117°15’E, 38°16’N, and the altitude is 7 m. The average annual precipitation is 627 mm, and the precipitation in flood season accounts for about 75% of the annual precipitation, which is prone to the phenomenon of ‘spring drought, summer flood and autumn hanging’. The average annual wind speed is 4.2 m/s, the average annual sunshine is 2755h, the average frost-free period is 194 d, and the average annual temperature is 12.1°C. The physical and chemical properties of the soil in the experimental plough layer are shown in **Table 1**, and the general situation of the experimental site (including the experimental plot layout and the distribution diagram of the coastal saline-alkali land in Hebei) is shown in **Figure 1**. The changes of the main meteorological factors during the growth period of soybean are shown in **Figure 2**.

**Table 1:** Soil physical and chemical properties.

Soil layer	Soil field capacity	Volumetric weight	Organic matter	Ph value	Water soluble salt	Electric conductivity
(cm)	(%)	(g/cm^3^)	(g/kg)		(‰)	(μs/cm)
0-20	17.15	1.48	9.38	8.7	4.1	1652
20-40	16.56	1.57	9.09	8.8	4.3	1867

### 2.2 Soybean and microorganisms

Although wild soybeans have high genetic diversity, they carry a large number of traits that are not conducive to agricultural production and are systematically eliminated under the goal of modern high-yield and high-efficiency breeding (Petereit et al., 2025). The main salt tolerance QTL carried by Jidou 12 was located on chromosome 3, with clear markers and significant effects. It showed the characteristics of salt tolerance, lodging resistance and antiviral disease in the coastal saline-alkali land of eastern Hebei. Therefore, Jidou 12 was selected as the tested soybean variety in this study (Shi et al., 2018).

*Bacillus subtilis* 8–32 and *Bradyrhizobium liaoningense* CCBAU05525, which were preserved by the Microorganism-Plant Interaction Laboratory of Hebei Agricultural University. *Bacillus subtilis* 8–32 was inoculated in LB medium for 24h, and the bacterial content was 3×10^8^ CFU/mL. *Bradyrhizobium liaoningense* CCBAU05525 was inoculated in YMA medium for 7 days, and the bacterial content was 7×10^8^ CFU/mL.

### 2.3 Experimental design

Before sowing, conventional fertilization was carried out, and the base fertilizer was applied with compound fertilizer N-P_2_O_5_-K_2_O (17-20-7) 375 kg/hm^2^. Sowing on June 28,2024, sowing on demand, applying microbial agent by seed dressing, row spacing 40 cm, plant spacing 9 cm. Test settings: CK for the control group (conventional fertilization). T1 is a single application of *Bacillus subtilis* treatment (conventional fertilization and application of 75L/hm^2^ *Bacillus subtilis* 8–32 agent). T2 was a single application of *Rhizobium* treatment (conventional fertilization and application of 75 L/hm^2^ *Bradyrhizobium liaoningense* CCBAU05525 agent). T3 was a co-inoculation treatment of *Bacillus subtilis* and *Rhizobium* (conventional fertilization, application of 75L/hm^2^ *Bacillus subtilis* 8–32 and 75L/hm^2^ *Bradyrhizobium liaoningense* CCBAU05525). The experiment consisted of four treatments, each treatment was repeated three times, and 12 experimental plots were randomly arranged. The plot area was 9m^2^ (3m×3m), and 1 m protective belt was set between the plots.

Harvested on October 12, the total fertility time was 110 days. It is divided into five growth stages: seedling stage, branching stage, flowering and podding stage, seed filling stage and maturity stage. The coastal saline-alkali land has no irrigation conditions, rain-fed dry farming, camera sowing according to soil moisture and rainfall conditions, and other management measures are the same as local farmers.

### 2.4 Determination of soybean yield and dry matter quality

On the second day after all soybeans were ripened, two quadrats with an area of 1 m^2^ were randomly selected in each treatment plot to determine the yield. By calculating the average yield of each treatment sample, the yield per hectare was converted. In addition, five plants were selected and marked in each plot, and the number of pods per plant, the number of seeds per pod and the weight of 100 seeds were measured to evaluate the yield components of soybean.

In each growth period of soybean, three healthy and representative plants were selected in each plot and divided into aboveground and underground parts. The plant samples were placed in an oven, first with 105 °C high temperature fixation for 30 minutes, and then adjusted to 80 °C drying to constant weight. At this time, the mass of each part was weighed with an electronic balance with an accuracy of 0.01 g, and the total dry matter mass was calculated.

### 2.5 Collection and preservation of soybean rhizosphere soil samples

On the second day after all soybeans were ripened, five soybean plants with the same growth vigor were randomly selected from each plot to collect rhizosphere soil samples. The steps are as follows: disinfect the shovel, dig down about 40 cm along the periphery of the soybean stem, pull up the plant with root and soil, and gently shake the root to collect the attached soil, put it in the corresponding sterile bag, and transport it to the laboratory at low temperature.

The collected soil samples were divided into three parts. One part was used to measure the soil moisture content by drying method, and the other part was dried, ground and sieved to measure the physical and chemical properties of rhizosphere soil. The last part was loaded into a sterile centrifuge tube, frozen in liquid nitrogen and stored in a -80 °Crefrigerator for analysis of bacterial community structure and soil enzyme activity.

### 2.6 Determination of physical and chemical properties and enzyme activity of soybean rhizosphere soil

The soil moisture content was determined by soil drying method, the soil pH value was determined by pen pH detector (PH8008), the soil conductivity was determined by conductivity meter (P902), the total amount of water-soluble salt in soil was determined by residue method, and the content of soil organic matter was determined by potassium dichromate volumetric method. The specific operation was based on “soil agrochemical analysis” compiled by Bosdan (Bao, 2000). Soil alkaline phosphatase activity was determined by disodium phenyl phosphate colorimetric method, sucrase activity was determined by DNS colorimetric method, urease activity was determined by indophenol blue colorimetric method, catalase activity was determined by potassium permanganate titration (Raiesi and Beheshti, 2014).

### 2.7 Method for determination of bacterial community in soybean rhizosphere soil

Ttal genomic DNA of soil bacterial communities was extracted using the E.Z.N.A.^®^ soil DNA kit (Omega Bio-tek). DNA quality was assessed via 1% agarose gel electrophoresis, and DNA concentration and purity were measured using a NanoDrop2000 spectrophotometer (Thermo Scientific). For PCR amplification, barcoded-specific primers were synthesized. To ensure data accuracy, low cycle numbers were used, and the cycle numbers were kept consistent across all samples. After preliminary experiments to determine the optimal conditions, formal experiments were conducted using TransGen AP221-02: TransStart Fastpfu DNA Polymerase and an ABI GeneAmp^®^ 9700 PCR instrument. Each sample included three replicates. The PCR products were mixed and detected via 2% agarose gel electrophoresis. The PCR products were then recovered using an AxyPrep DNA gel recovery kit, eluted with Tris-HCl, and retested with 2% agarose gel electrophoresis.

For fluorescence quantification, the PCR products were quantified using the QuantiFluor™ -ST fluorescence quantification system (Promega) based on the preliminary results of electrophoresis, and the samples were mixed in proportion to the sequencing requirements. During library construction, adapter sequences were added to the target regions by PCR. The PCR products were recovered and eluted with Tris-HCl buffer, followed by 2% agarose gel electrophoresis detection. Single-stranded DNA fragments were generated by sodium hydroxide denaturation using the TruSeqTM DNA Sample Prep Kit. In sequencing, the adapter sequences of the DNA fragments were complementary to the base sequences embedded in the chip and were fixed on the chip. The base sequences fixed on the chip were used as primers for PCR synthesis of the target DNA fragments. After denaturation and annealing, the other end of the DNA fragments on the chip randomly complemented another nearby primer and was also fixed, forming a “bridge”. PCR amplification generated DNA clusters. After linearization of the DNA amplicons, modified DNA polymerase and fluorescently labeled dNTPs were added. One base was incorporated per cycle. The reaction plate surface was scanned with a laser to read the nucleotide types added to each template sequence in a single reaction cycle. The “fluorescent groups” and “terminator groups” were chemically cleaved to restore the 3’ end stickiness, allowing further nucleotide polymerization. The fluorescence signals collected in each cycle were counted to determine the sequence of the template DNA fragments.

### 2.8 Data analysis

Experimental data were arranged by Microsoft Excel 2019 and statistical analysis including significance test at *P* < 0.05 level and univariate analysis of variance by SPSS 27.0. Charts were drawn by using Origin 2023.

## 3 Results

### 3.1 Effects of co-inoculation on yield and dry matter accumulation of soybean in saline-alkali land

#### 3.1.1 Effects of co-inoculation on soybean yield and yield components in saline-alkali land

As shown in **Table 2**, it can boost soybean yield by bioinoculant application. Soybean yield is 2696.48 kg/hm² in CK treatment. The yield increased by 7.58% to 18.03% in bioinoculant application, compared with the CK treatment. Co-inoculation with *Bacillus subtilis* and *Bradyrhizobium liaoningense* CCBAU05525 (T3) was most effective, achieving the highest yield of 3182.67 kg/hm². It was a significant 5.2% and 9.7% increase compared to T1 and T2, respectively. The yield is 3024.52 kg/hm² and 2900.99 kg/hm² in T1 (*Bacillus subtilis* alone) and T2 (*Rhizobium* alone) treatment respectively, the difference between them was not significant (*P*>0.05). Seeds per pod and 100-seed weight increase 5.32%-18.62% and 0.40%-2.68% respectively by bioinoculant application compared with the control. Seeds per pod and 100-seed weight were highest in Co-inoculation treatment, its values was 2.23 and 22.99 g. The single application of *Bacillus subtilis* 8–32 and *Bradyrhizobium liaoningense* CCBAU05525 was helpful to increase the number of pods per plant. There was no significant difference in the number of pods per plant inoculated with *Bacillus subtilis* and *Rhizobium*. The increase of co-inoculation yield of *Bacillus subtilis* and *Rhizobium* was mainly due to the increase of grain number per pod and 100-grain weight.

**Table 2:** Effects of co-inoculation of *Bacillus subtilis* and *Rhizobium* on soybean yield and its components in saline-alkali soil.

Numbering	Number of pods per plant (a)	Number of seeds per pod (a)	Hundred grain weight (g)	Production (kg/hm^2^)
CK	26.60 ± 1.06a	1.88 ± 0.03d	22.39 ± 0.10c	2696.48 ± 81.17c
T1	26.67 ± 0.95a	2.05 ± 0.05c	22.71 ± 0.15b	3024.52 ± 96.20b
T2	27.07 ± 0.81a	1.98 ± 0.03b	22.48 ± 0.03c	2900.99 ± 67.26b
T3	25.80 ± 1.11a	2.23 ± 0.03a	22.99 ± 0.13a	3182.67 ± 53.55a

CK for the control group. T1 is a single application of *Bacillus subtilis* treatment. T2 was a single application of *Rhizobium* treatment. T3 was a co-inoculation treatment of *Bacillus subtilis* and *Rhizobium*. Different lowercase letters after the same column numbers in the table indicate that the difference between different treatments of the same index reaches a significant level (*P* < 0.05).

#### 3.1.2 Effect of co-inoculation on dry matter accumulation of soybean in saline-alkali land

**Figure 3:**
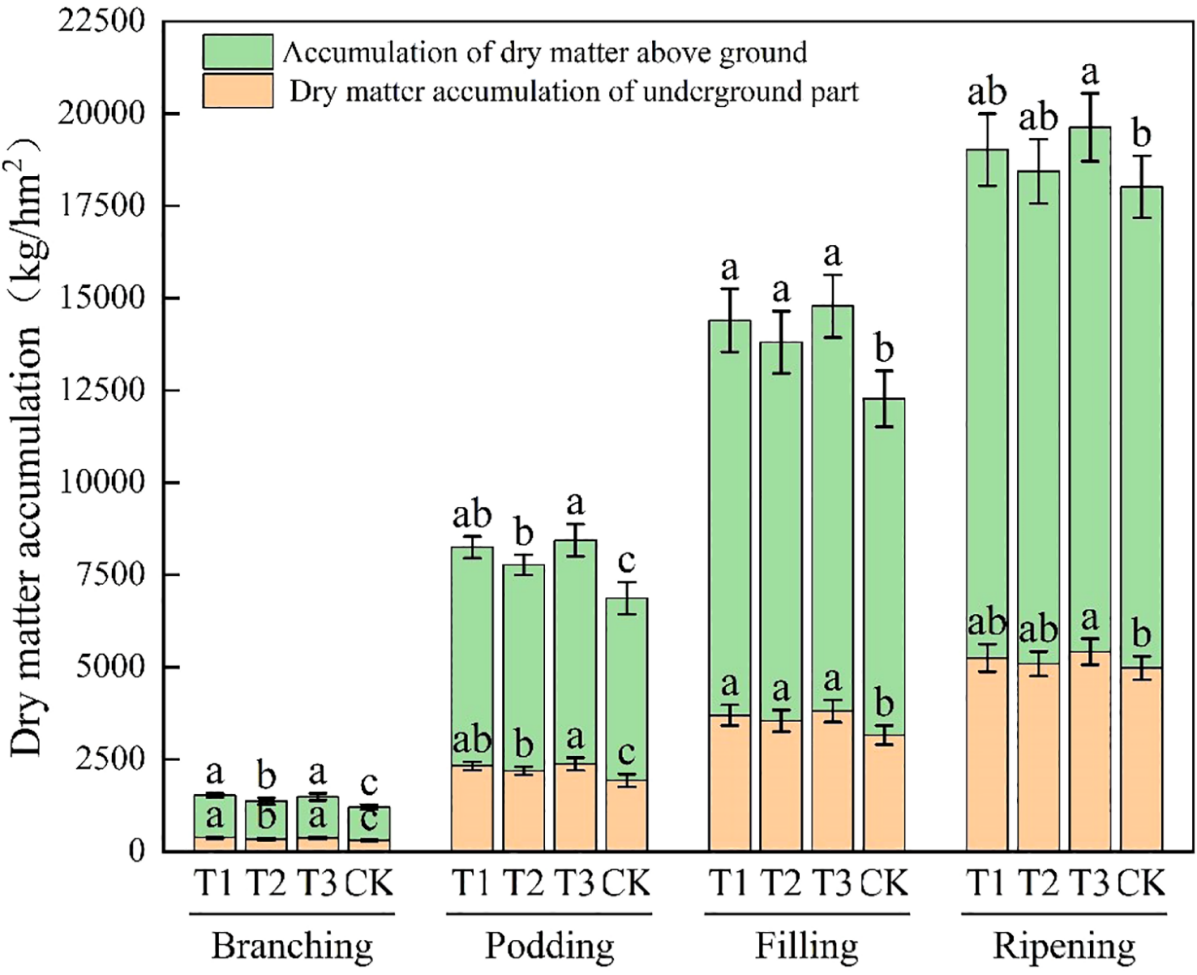
Dry matter accumulation of soybean at different growth stages. CK for the control group. T1 is a single application of *Bacillus subtilis* treatment. T2 was a single application of *Rhizobium* treatment. T3 was a co-inoculation treatment of *Bacillus subtilis* and *Rhizobium*.

It can be concluded from **Figure 3** that with the advance of the growing stage, the dry matter accumulation in the above-ground part of soybean shows an increasing trend. During the branching stage, the rate of increase is relatively slow. The accumulation amounts of T1 and T3 treatments reach 1144.39 kg/hm² and 1114.63 kg/hm² respectively, which are significantly higher than those of T2 and CK treatments (*P* < 0.05). In the flowering-podding, grain-filling, and maturation stages, the growth rate speeds up. In the flowering-podding stage, the accumulation amount of T3 treatment is 6064.89 kg/hm², which is significantly higher than that of T2 and CK (*P* < 0.05). During the grain-filling stage, the values of T1, T2, and T3 treatments are 10696.73 kg/hm², 10257.69 kg/hm², and 10976.39 kg/hm² respectively, all of which are significantly higher than that of the CK treatment (*P* < 0.05). In the maturation stage, the accumulation amount of T3 treatment is 14206.18 kg/hm², which is significantly higher than that of CK treatment (*P* < 0.05). The dry matter accumulation in the above-ground part of soybean increase by application of *Bacillus subtilis* or *Rhizobium* alone. However, it is the most remarkable effect for the combined application of both.

The dry matter accumulation in the underground part of soybean also increases with the growing stage. During the branching stage, the accumulation is relatively low, The dry matter accumulation in the underground part of soybean of T1 and T3 treatments is 378.26 kg/hm² and 368.96 kg/hm², both significantly higher than that of T2 and CK (*P* < 0.05). In the flowering-podding, grain-filling, and maturation stages, the accumulation increases more rapidly. In the flowering-podding stage, the accumulation of T3 treatment is 2374.36 kg/hm², surpassing that of T2 and CK significantly (*P* < 0.05). In the grain-filling stage, the accumulation of T1, T2, and T3 treatments is 3692.70, 3542.94, and 3803.69 kg/hm², all outperforming that of CK treatment significantly (*P* < 0.05). In the maturation stage, the accumulation of T3 treatment attains 5416.577 kg/hm², significantly higher than that of CK treatment (*P* < 0.05). Both single *Bacillus subtilis* and single *Rhizobium* application boost underground dry matter accumulation significantly. However, their combined application works best.

As the growing stage advances, the total dry matter accumulation in soybean gradually increases. In the branching stage, the growth rate is relatively slow. The accumulation amounts of T1 and T3 treatments are 1522.65 kg/hm² and 1483.59 kg/hm², which are significantly higher than those of T2 and CK treatments by 8.87% to 26.31% (*P* < 0.05). In the flowering-podding, grain-filling, and maturation stages, the growth rate accelerates. In the flowering-podding stage, the accumulation amounts of T3 treatment reaches 8439.25 kg/hm², which is significantly higher than that of T2 and CK treatment by 8.62% to 22.96% (*P* < 0.05). During the grain-filling stage, the values of T1, T2, and T3 treatments are 14389.43 kg/hm², 13800.63 kg/hm², and 14780.08 kg/hm², all of which are significantly higher than that of CK (*P* < 0.05). In the maturation stage, the T3 treatment shows the highest value of 19622.75 kg/hm², which is significantly higher than that of CK by 8.93% (*P* < 0.05). There is a significant difference between T3 and CK treatments (*P* < 0.05), but no significant difference between T3 and the other treatments (*P*>0.05).The application of either *Bacillus subtilis* or *Rhizobium* alone can enhance the total dry matter accumulation. However, the combined application of both shows the best results.

Compared to CK, the application of bioinoculants alone or in combination can increase the total dry matter accumulation in soybean. Among these, single *Bacillus subtilis* application is superior to *Rhizobium*, and the combined application of both shows the best performance. Since dry matter accumulation, which is closely related to the total organic matter of the plant and grain yield, represents the photosynthetic products, the application of bioinoculants can enhance grain yield by increasing dry matter accumulation.

### 3.2 Effects of co-inoculation on physicochemical properties of soybean rhizosphere soil in saline-alkali land

As shown in **Table 3**, different inoculant treatments have varying effects on the physicochemical properties of soybean rhizosphere soil. The soil moisture content of T1 and T3 treatments reaches 16.14% and 16.32%, significantly higher than CK (4.5%–5.6%), but no significant difference is found between the bioinoculant treatments (*P*>0.05). The pH values of T1, T2, and T3 treatments are 8.37, 8.43, and 8.33, all significantly lower than that of CK (1.6%–2.8% lower), but again, no significant difference exists among the inoculant treatments (*P*>0.05). The electrical conductivity of T1 and T3 is 1340.33 μs/cm and 1301.33 μs/cm, marking an 8.3%–11.0% decrease compared with that of CK, yet the difference electrical conductivity of between T1 and T3 is not significant (*P*>0.05). The water soluble salt of T1 and T3 is 3.52‰ and 3.5‰, representing a 4.9%–5.4% reduction compared to that of CK. Notably, T2 shows no significant difference from CK (*P*>0.05). The organic matter content of T1, T2, and T3 is 13.23 g/kg, 12.81 g/kg, and 13.45 g/kg, a 6.3%–11.6% increase over that of CK, but no significant difference is observed among the inoculant treatments (*P*>0.05).

**Table 3:** Effects of co-inoculation of *Bacillus subtilis* and *Rhizobium* on physical and chemical properties of soybean rhizosphere soil in saline-alkali land.

Numbering	Soil moisture content (%)	Ph value	Electric conductivity (μs/cm)	Water soluble salt (‰)	Organic matter (g/kg)
CK	15.45 ± 0.14b	8.57 ± 0.06a	1462.33 ± 46.5a	3.7 ± 0.09a	12.05 ± 0.46b
T1	16.14 ± 0.53a	8.37 ± 0.06b	1340.33 ± 54.1b	3.52 ± 0.1b	13.23 ± 0.42a
T2	15.93 ± 0.19ab	8.43 ± 0.06b	1368.33 ± 78.95ab	3.7 ± 0.07a	12.81 ± 0.14a
T3	16.32 ± 0.23a	8.33 ± 0.06b	1301.33 ± 33.56b	3.5 ± 0.06b	13.45 ± 0.31a

CK for the control group. T1 is a single application of *Bacillus subtilis* treatment. T2 was a single application of *Rhizobium* treatment. T3 was a co-inoculation treatment of *Bacillus subtilis* and *Rhizobium*. Different lowercase letters after the same column numbers in the table indicate that the difference between different treatments of the same index reaches a significant level (*P* < 0.05).

Overall, the soil moisture, pH, electrical conductivity, water soluble salt, and organic matter of T1 and T3 treatments significantly improve in soybean rhizosphere soil on saline-alkali land, which alleviate stress, optimize nutrient availability, and enhance soil structure. This creates a favorable root environment that promotes the growth of lateral roots and root hairs. Consequently, photosynthetic efficiency, root system function, and grain filling are improved, leading to a significant increase in soybean yield.

### 3.3 Effect of co-inoculation on enzyme activity in rhizosphere soil of soybean in saline-alkali land

**Figure 4:**
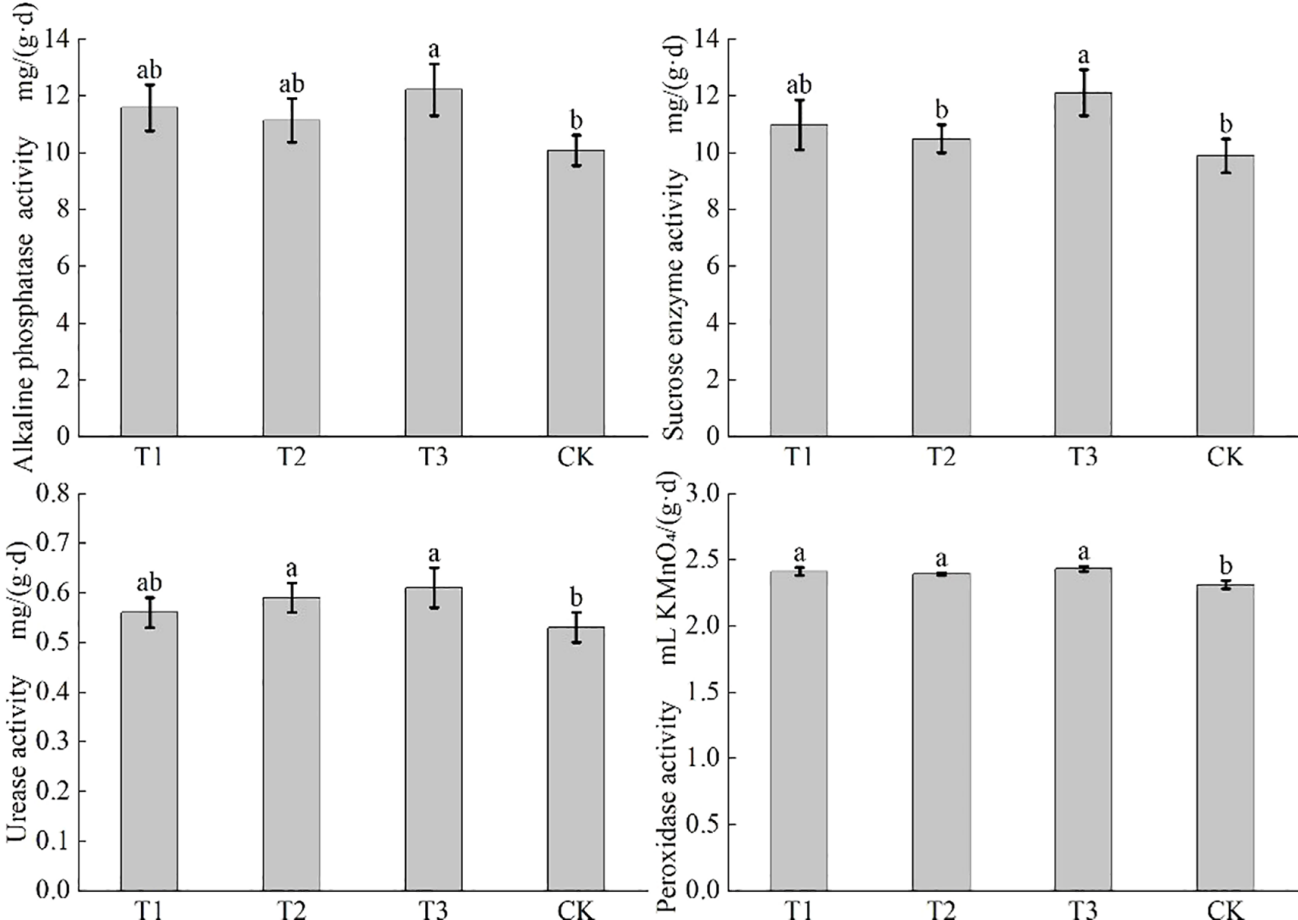
Effects of co-inoculation of *Bacillus subtilis* and *Rhizobium* on enzyme activities in rhizosphere soil of soybean in saline-alkali land. The effects of co-inoculation of *Bacillus subtilis* and *Rhizobium* on alkaline phosphatase activity, sucrase activity, urease activity and catalase activity in rhizosphere soil of soybean in saline-alkali land were expressed in turn. CK for the control group. T1 is a single application of *Bacillus subtilis* treatment. T2 was a single application of *Rhizobium* treatment. T3 was a co-inoculation treatment of *Bacillus subtilis* and *Rhizobium*.

**Figure 4** shows that T3 treatment remarkably elevated soil alkaline phosphatase activity to 12.22 mg/(g·d), a 14.9% increase compared with that of CK (*P* < 0.05), yet showed no significant difference from other treatments. T3 also enhanced sucrose enzyme activity to 12.11 mg/(g·d), up by 15.4% and 22.4% compared to that of T2 and CK (*P* < 0.05), but not significantly different from T1. The urease activity of T2 and T3 was 0.59 and 0.61 mg/(g·d), marking an 11.3%-15.1% increase from CK (*P* < 0.05), with no significant difference from T1. The peroxidase activity of T1, T2, and T3 was 2.41 mL KMnO_4_/(g·d), 2.39 mL KMnO_4_/(g·d), and 2.43 mL KMnO_4_/(g·d), all significantly higher than that of CK (*P* < 0.05), but with no significant differences among inoculant treatments. In summary, T3 was most effective in boosting soil enzyme activities, especially for sucrose enzyme and alkaline phosphatase, indicating a strong promotion of soil fertility and nutrient transformation.

Alkaline phosphatase activity improves by application of either single *Bacillus subtilis* or *Rhizobium*, with single *Bacillus subtilis* being more effective, which of co-inoculation (T3) achieved the most significant boost. Sucrose enzyme and urease activities of T3 are the highest. Peroxidase activity of all bio inoculant treatments significantly rose. It was T3 that the optimal treatment for enhancing the four soil enzyme activities. The synergy of alkaline phosphatase and urease meets soybean’s phosphorus and nitrogen needs, reducing nutrient competition. Sucrose enzyme facilitates organic matter decomposition, creating a virtuous cycle, while peroxidase alleviates oxidative damage. Together with sucrose enzyme, which improves soil structure, it boosts stress resistance. The co-inoculation of *Bacillus subtilis* and *Rhizobium* enhances nutrient release, strengthens microbial functions, and mitigates stresses. It significantly improves the rhizosphere environment for sustainable soybean yield increases.

### 3.4 Effects of co-inoculation on bacterial community composition in rhizosphere soil of soybean in saline-alkali land

#### 3.4.1 OTU analysis and Venn diagram analysis of soil bacteria

**Figure 5:**
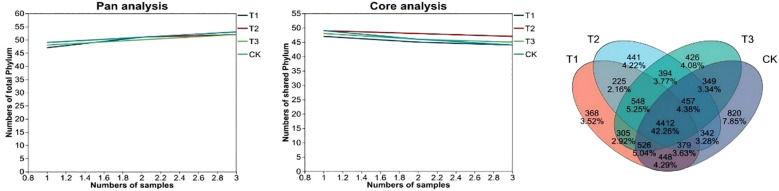
Pan and Core species analysis diagram and Venn diagram of different treatments. CK for the control group. T1 is a single application of *Bacillus subtilis* treatment. T2 was a single application of *Rhizobium* treatment. T3 was a co-inoculation treatment of *Bacillus subtilis* and *Rhizobium*.

Microbial taxonomy and abundance in environmental samples are obtained by OTU analysis clusters, transforms sequencing reads and matches them to species databases. Whether the sample size is sufficient is assessed by Pan-and core species analysis (**Figure 5**). Pan OTU is the sum of all sample OTUs, showing total OTU number changes as sample number increases; Core OTU is the shared OTUs across all samples, reflecting the change in common OTUs with more samples. Results show that bioinoculants can change the composition and diversity of soil bacterial communities, and the four treatment’s sample sizes were adequate.

A Venn diagram (**Figure 5**) can intuitively count the shared and unique species (e.g., OTUs) among multiple groups, thereby reflecting the similarity and overlap in species composition. During analysis, OTUs at a similarity level of 97% or other taxonomic levels are commonly used. As shown in **Figure 5**, the OTU composition and overlap vary among different treatments. T1 and T2 share 5564 OTUs, T2 and T3 share 5811, and T3 and CK share 5744. The unique bacterial species counts of T1, T2, T3, and CK are 368, 441, 426, and 820, respectively. Overall, bioinoculant application reduces the OTU count. It achieves the goal of “reducing OTU numbers while maintaining yield,” showing remarkable effects in saline-alkali land.

#### 3.4.2 Analysis of α diversity of soil bacterial community under different treatments

**Table 4** shows the richness and diversity indices of samples at the 97% similarity level. The Coverage index of all treatments was between 0.97 and 0.99 showing that the sequencing data accurately reflects sample diversity. The Sobs index indicates community richness, with higher values signifying greater richness. The Sobs index of CK is higher than that of T1 and T2. The Shannon index assesses microbial diversity and alpha diversity of bacterial communities, where higher values indicate more diverse communities. The Shannon index of T1 is lower than that of the other treatments. A higher Simpson index means lower diversity. The Simpson index of T1 is higher than that of the other treatments. The Ace and Chao indices estimate the number of OTUs in bacterial communities, with higher values indicating greater richness. There were no significant differences in Ace and Chao indices among treatments. In brief, the combined *Bacillus subtilis* and *Rhizobium* (T3) optimized the α-diversity of rhizosphere soil bacterial communities, which is more favorable for soybean growth. As the key interface for soybean-soil interaction, well structure of rhizosphere bacterial communities can promote root growth, enhance nutrient and water uptake, suppress pathogens, reduce diseases, and boost soybean yield.

**Table 4:** Effects of co-inoculation of *Bacillus subtilis* and *Rhizobium* on α diversity of bacterial community in rhizosphere soil of soybean in saline-alkali land.

Numbering	Sobs index	Shannon diversity index	Simpson index	Ace indices	Chao index	Coverage index
CK	5433.00 ± 225.89a	7.15 ± 0.08a	0.0034 ± 0.00106b	6476.24 ± 256.55a	6384.50 ± 214.49a	0.9808 ± 0.00131a
T1	4959.33 ± 189.23b	6.46 ± 0.19b	0.0312 ± 0.01114a	5921.32 ± 182.91a	5857.37 ± 165.90a	0.9806 ± 0.0019a
T2	4990.33 ± 132.30b	7.06 ± 0.12a	0.0048 ± 0.0018b	5967.79 ± 282.12a	5911.85 ± 289.99a	0.9787 ± 0.00346a
T3	5160.67 ± 275.25ab	7.09 ± 0.03a	0.0036 ± 0.00024b	6227.70 ± 384.17a	6153.75 ± 377.11a	0.9793 ± 0.00087a

CK for the control group. T1 is a single application of *Bacillus subtilis* treatment. T2 was a single application of *Rhizobium* treatment. T3 was a co-inoculation treatment of *Bacillus subtilis* and *Rhizobium*. Different lowercase letters after the same column numbers in the table indicate that the difference between different treatments of the same index reaches a significant level (*P* < 0.05).

#### 3.4.3 Analysis of soil bacterial community composition under different treatments

**Figure 6:**
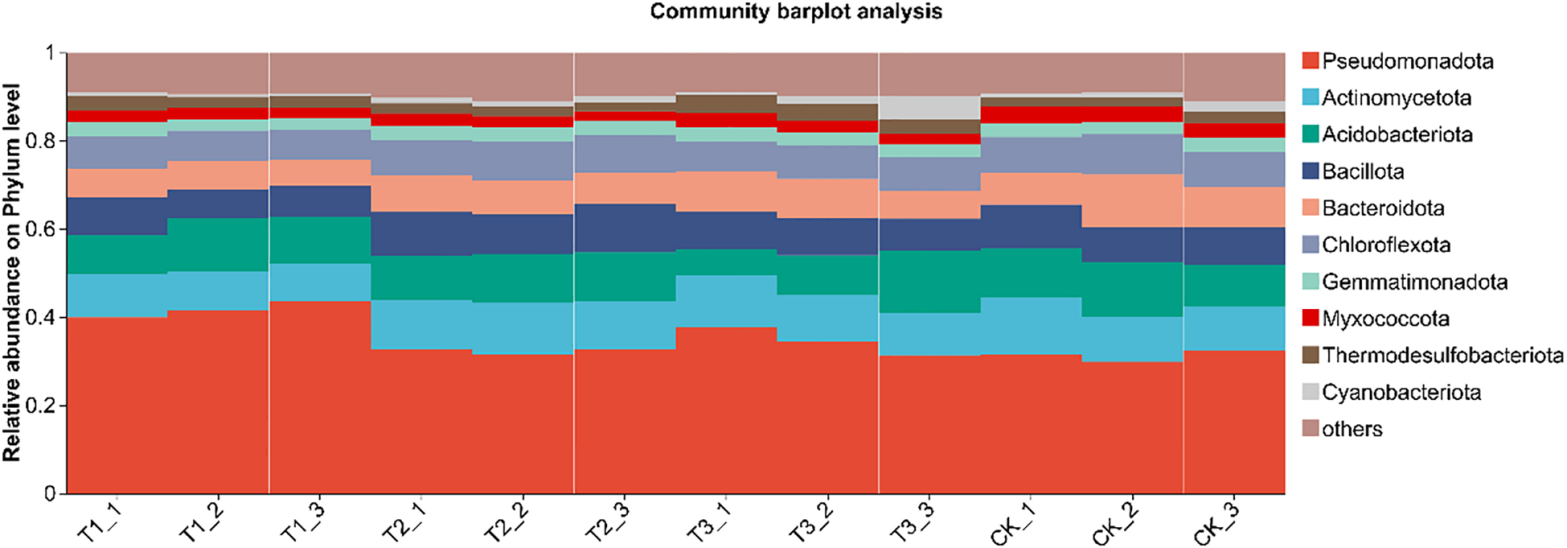
Species composition of soil bacteria at phylum level under different treatments. CK for the control group. T1 is a single application of *Bacillus subtilis* treatment. T2 was a single application of *Rhizobium* treatment. T3 was a co-inoculation treatment of *Bacillus subtilis* and *Rhizobium*.

After identifying the species corresponding to each OTU, multiple OTUs can be linked to the same species. Hence, OTUs classified as the same species were merged to analyze bacterial community composition across different treatments. The phylum level was focused on in paper. Histogram was created for the top 10 species in each treatment, with the rest categorized as “others.” Relative abundance analysis was conducted based on species proportions, with results presented in **Figure 6**. In the phylum level, soil bacterial communities of all treatments were predominantly composed of Pseudomonadota (31.27%–41.67%), with Actinomycetota (9.10%–11.30%), Acidobacteriota (9.64%–10.97%), and Bacillota (7.38%–9.96%) as secondary components. Compared with the other treatments, T1 significantly elevated the relative abundance of Proteobacteria (*P* < 0.05), whereas no statistical differences were observed among T2, T3 and CK (*P*>0.05). The proportion of Actinobacteria in T2 was significantly higher than that in T1 (*P* < 0.05), but remained comparable to T3 and CK (*P*>0.05). No significant inter-treatment variations were detected for Acidobacteria (*P*>0.05). Firmicutes were markedly enriched in T2 relative to T1 (*P* < 0.05), yet similar between T2, T3 and CK (*P*>0.05). Collectively, bio-inoculation reshaped the soil bacterial community composition at the phylum level, fostering a diverse and robust microbial assemblage that underpins high soybean yields in saline–alkali soils.

**Figure 7:**
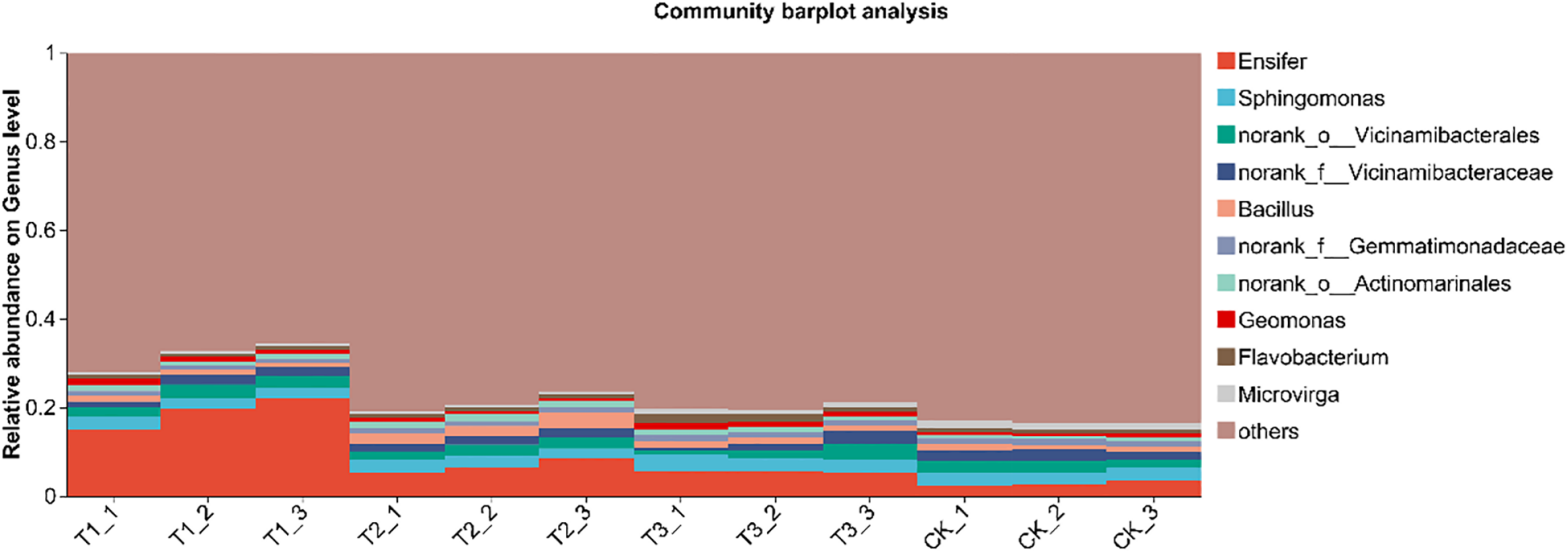
Species composition of soil bacteria at genus level under different treatments. CK for the control group. T1 is a single application of *Bacillus subtilis* treatment. T2 was a single application of *Rhizobium* treatment. T3 was a co-inoculation treatment of *Bacillus subtilis* and *Rhizobium*.

In the genus-level analysis, a histogram was created for the top 10 species in each treatment, with the rest categorized as “others.” Relative abundance analysis was conducted based on species proportions (**Figure 7**). Results showed that Ensifer dominated (2.81%–18.92%), followed by Sphingomonas(2.57%–3.28%) and Bacillus (1.11%–2.73%). Compared with the other treatments, T1 markedly enriched the genus Ensifer (*P* < 0.05), while no significant differences were detected among T2, T3, and CK (*P*>0.05). T3 significantly elevated the relative abundance of Sphingomonas relative to T1 and T2 (*P* < 0.05). Bacillus was most abundant in T2, surpassing all other treatments (*P* < 0.05), and remained comparable among T1, T3, and CK (*P*>0.05). Overall, bio-inoculation restructured the soil bacterial community at the genus level, fostering a beneficial consortium that underpins the high-yield potential of soybean in saline–alkali soils.

**Figure 8:**
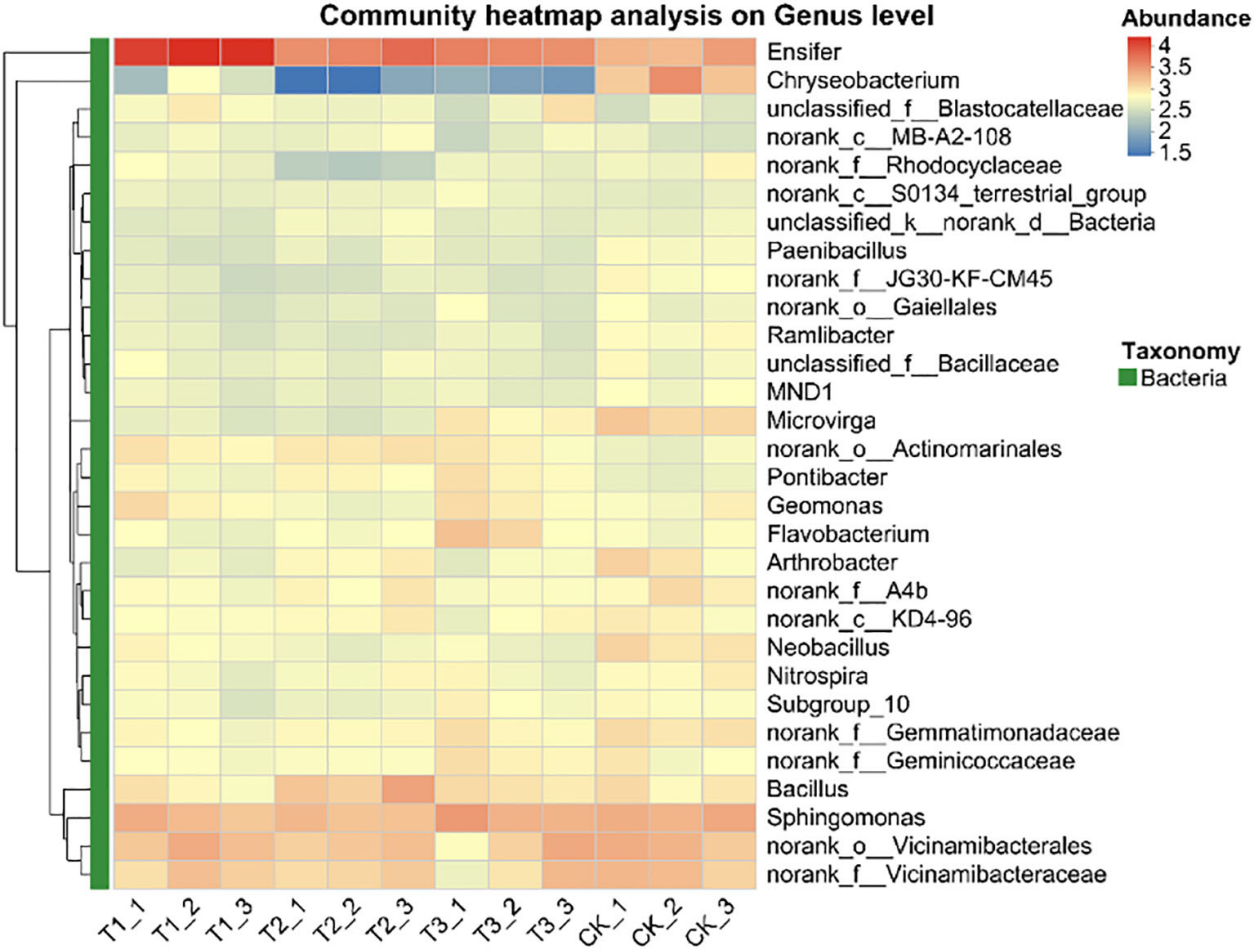
Relative abundance of soil bacteria at genus level under different treatments. CK for the control group. T1 is a single application of *Bacillus subtilis* treatment. T2 was a single application of *Rhizobium* treatment. T3 was a co-inoculation treatment of *Bacillus subtilis* and *Rhizobium*.

The top 30 species in abundance at the genus level was identified by analysis soil samples from different treatments and the mean abundance of replicate samples within each group was calculated, a heatmap was created by using this data and sample information (**Figure 8**). The results showed that Ensifer and Sphingomonas were the dominant bacterial genera in all treatments and soil layers. Compared to non - inoculated controls, the inoculant - applied treatments altered the relative abundance of some genera but maintained overall abundance balance.

#### 3.4.4 Relationship between soil bacterial samples and species under different treatments

**Figure 9:**
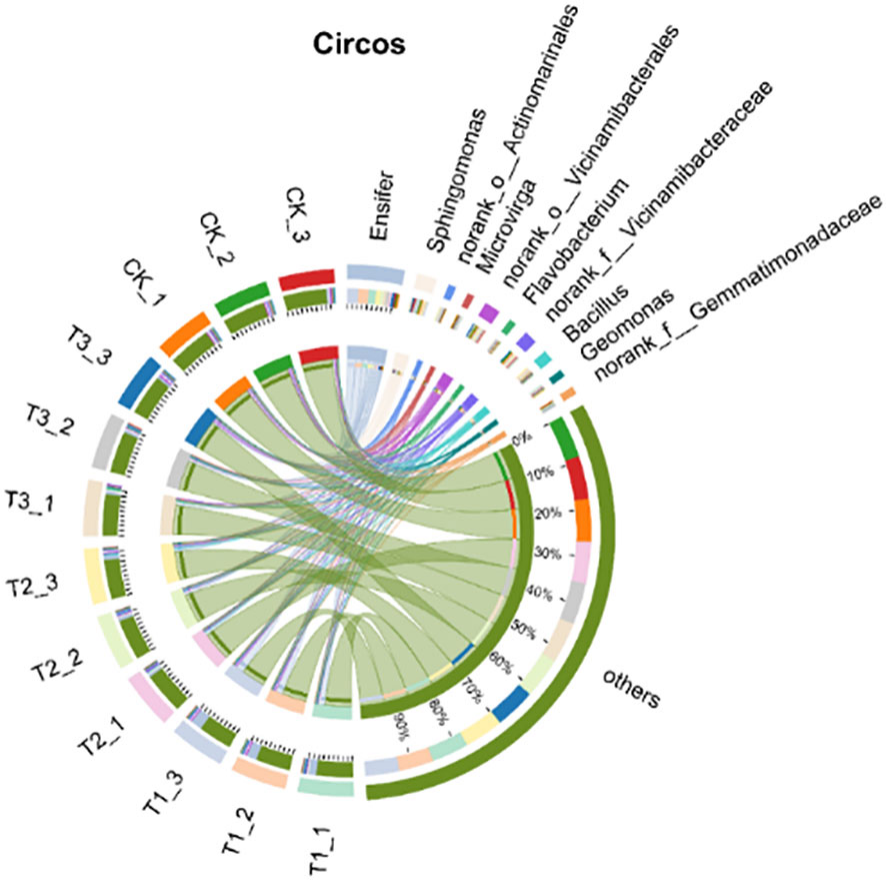
The relative abundance of bacterial flora in soybean rhizosphere soil under different treatments. CK for the control group. T1 is a single application of *Bacillus subtilis* treatment. T2 was a single application of *Rhizobium* treatment. T3 was a co-inoculation treatment of *Bacillus subtilis* and *Rhizobium*.

**Figure 9** shows the top 10 bacterial genera in relative abundance of the different treatments in the rhizosphere soil of soybeans including Ensifer (2.33%-18.67%), Sphingomonas (7.67%-9.67%), Bacillus (5.67%-14.00%), and others. Ensifer’s relative abundance of T1 treatment is significantly higher than that of other treatments (*P* < 0.05), while the relative abundance of other genera shows no clear difference. There is a synergistic effect among the genera, and the microbial support network for high and stable yield of soybean is constructed, which promotes the sustainable high yield of soybean.

#### 3.4.5 PCoA and cluster analysis

**Figure 10:**
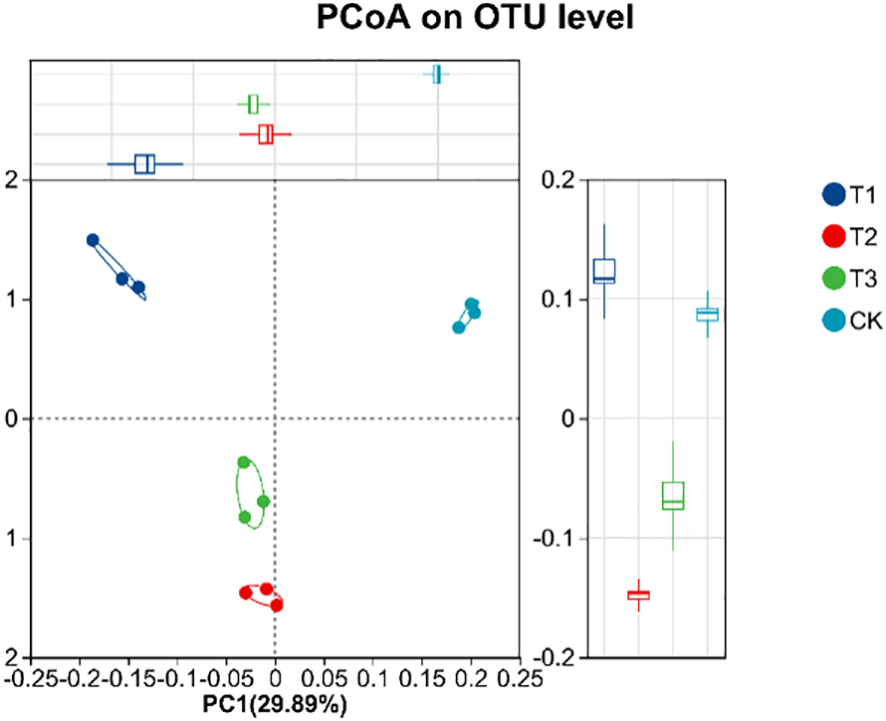
PCoA diagram of soil bacterial community under different treatments. CK for the control group. T1 is a single application of *Bacillus subtilis* treatment. T2 was a single application of *Rhizobium* treatment. T3 was a co-inoculation treatment of *Bacillus subtilis* and *Rhizobium*.

**Figure 11:**
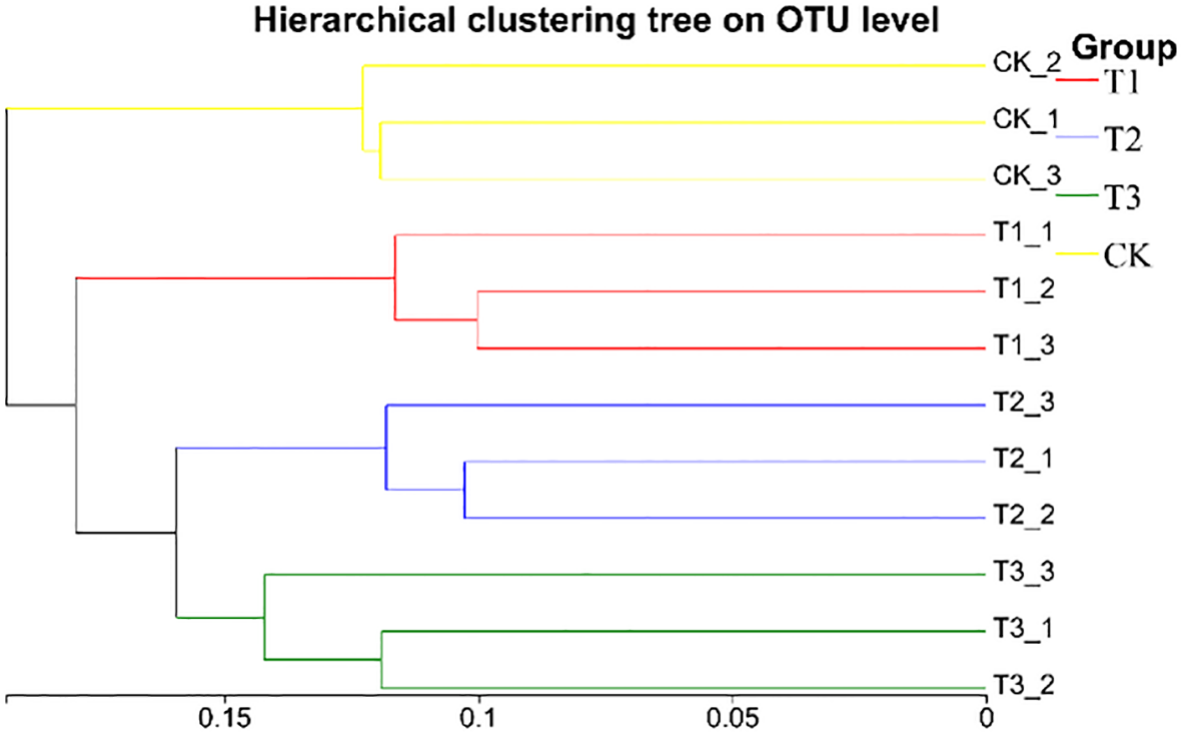
Hierarchical cluster analysis of soil bacterial samples in different treatments. CK for the control group. T1 is a single application of *Bacillus subtilis* treatment. T2 was a single application of *Rhizobium* treatment. T3 was a co-inoculation treatment of *Bacillus subtilis* and *Rhizobium*.

Beta diversity of soil bacterial community was analyzed by using PCoA. **Figure 10** showed that there are significant differences in soil sample groups. In the 4 groups of soil samples, the bacterial community in the group could be well polymerized in the PC1 axis (29.89%), and the distance between the sample points in different groups was relatively large, indicating that the composition of bacterial community changed greatly after the application of biological agents. The structure of community of T3 group was relative concentration and difference from CK group, a change caused by co-inoculation with *Bacillus subtilis* and *Rhizobium*, which increased beneficial soil microorganism numbers and activity, positively affecting soybean yield. Meanwhile, the UPGMA cluster analysis (**Figure 11**) also showed that the soil bacterial of T1, T2, T3, and CK samples had small within - group differences but large between - group differences. The soil bacterial community structures of T1, T2, and T3 were highly similar, but the CK group showed significant divergence from others. Based on soil bacterial OTUs, the PCoA results showed that soil bacterial communities in different treatments had significant differences, which were closely related to soybean yield. The community structure of T3 group was relative stability, which had a positive impact on soybean yield.

#### 3.4.6 Prediction analysis of soil bacterial population function

**Figure 12:**
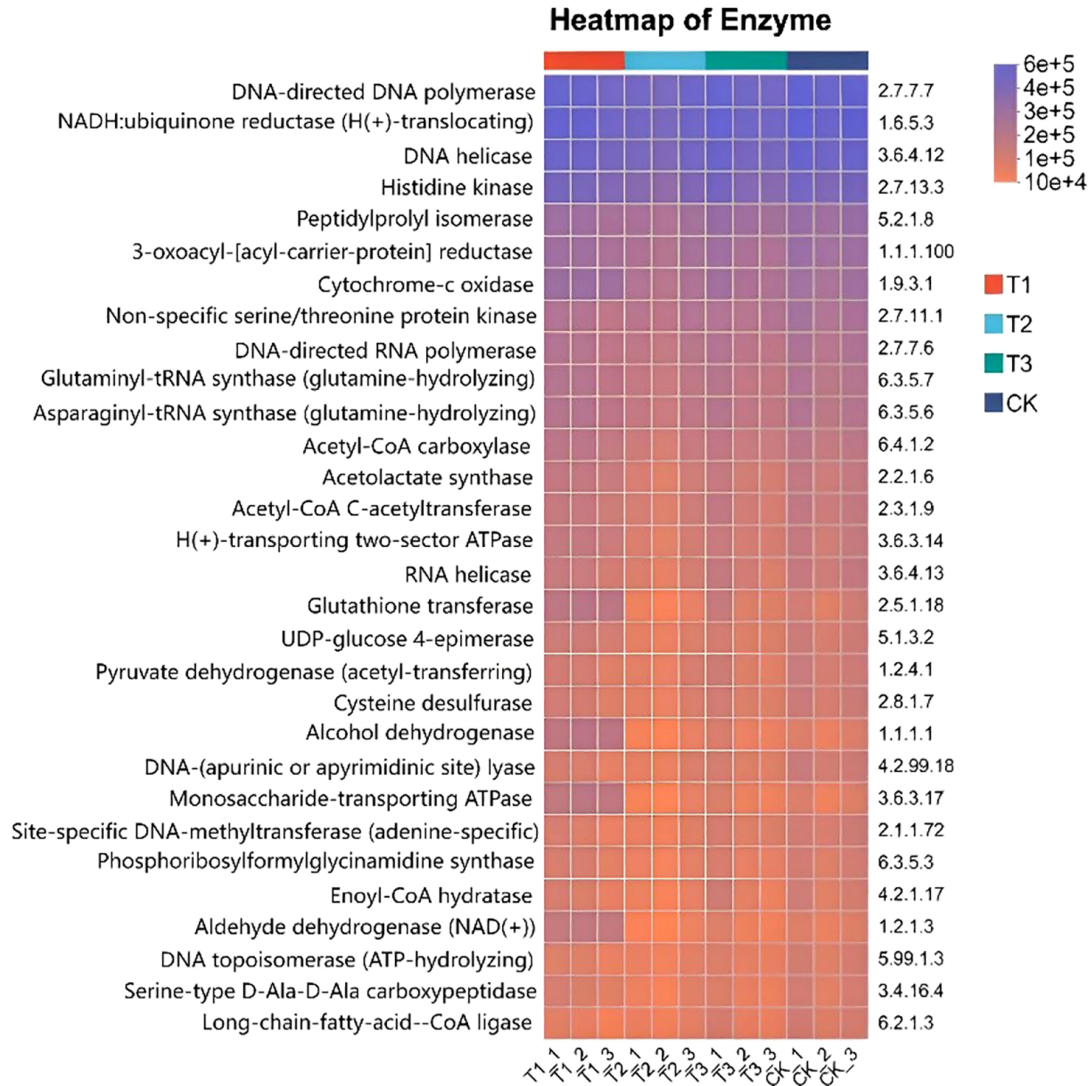
KEGG function prediction of soil bacterial flora in different treatments. CK for the control group. T1 is a single application of *Bacillus subtilis* treatment. T2 was a single application of *Rhizobium* treatment. T3 was a co-inoculation treatment of *Bacillus subtilis* and *Rhizobium*.

The 16S rRNA feature sequences were analyzed by using the PICRUSt2 tool in paper, evolutionary tree was constructed, and community pathways were predicted (**Figure 12**). The most of enzyme activity levels of T3 treatment was higher and the color was darker. The enzyme activity of the remaining treatments was relatively low. In most enzymes, the activity of T1 treatment was lower, the color was lighter, and the activity was close to 1 × 10^4^. The enzyme activity of T3 treatment was mainly concentrated between 4 × 10^5^ and 6 × 10^5^, and the color was darker. The enzyme activity of T2 and CK were intermediate between T1 and T3. A variety of enzyme activities of T3 treatment increased, such as DNA-directed DNA polymerase to accelerate cell division, DNA-directed RNA polymerase to accelerate gene transcription, NADH: ubiquinone reductase to enhance energy metabolism, and Peptidylprolyl isomerase to ensure the correct folding of proteins, all of which are crucial for soybean growth and yield. There is a positive impact on soil enzyme activity in T3 treatment, which is favorable for maintaining the balance of the enzyme system and stabilizing soil nutrient cycling. In addition, the research also involves amino acid metabolism, carbohydrate metabolism and other fields. These results provide an important basis for understanding soil bacterial community function and ecosystem function.

### 3.5 Interaction between soil indicators and yield

**Figure 13:**
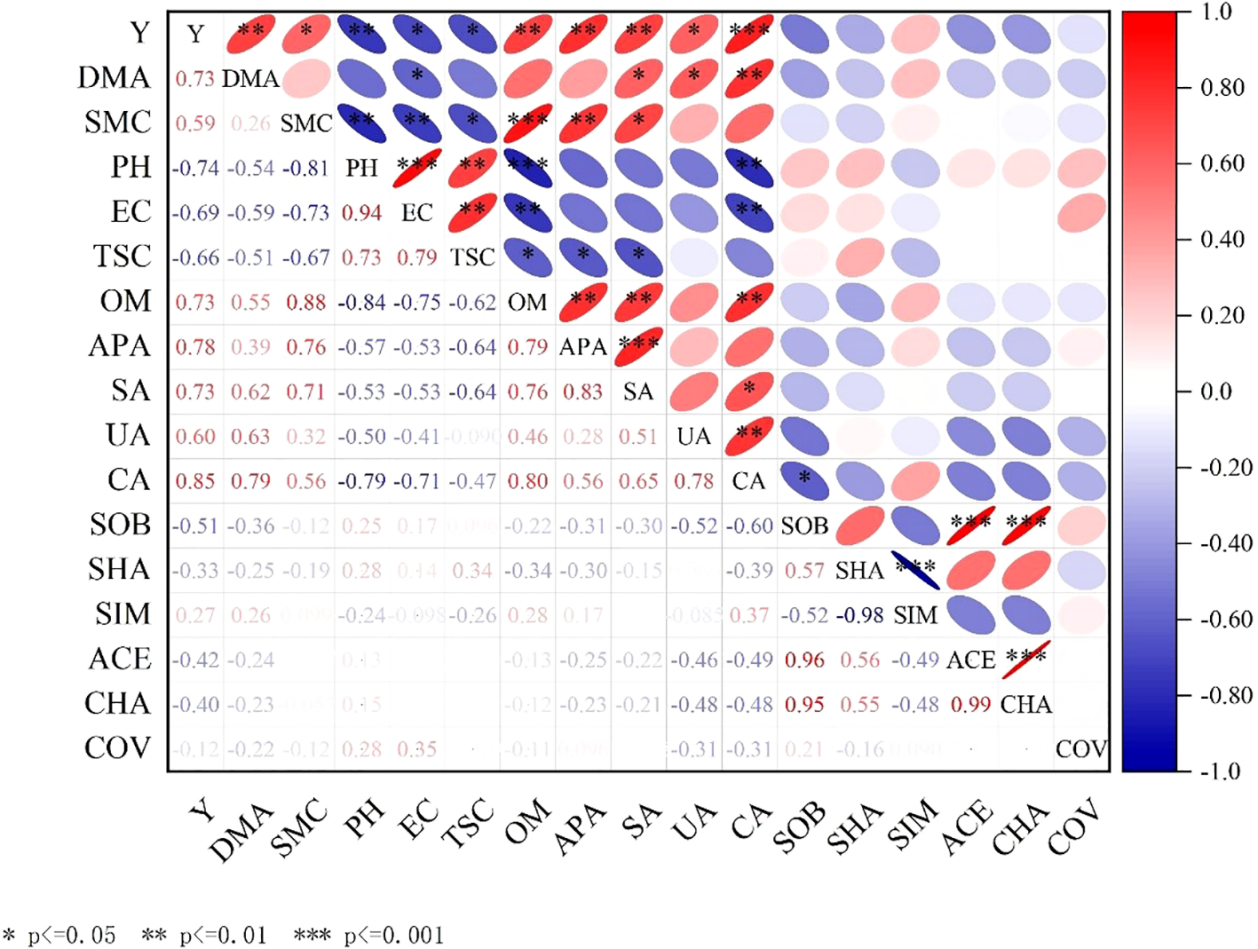
The interaction between soil index and yield. Y, represents grain yield; DMA, represents dry matter accumulation; SMC, represents soil moisture content; PH, represents soil pH; EC, represents electrical conductivity; TSC, represents water soluble salt; OM, represents organic matter; APA, represents alkaline phosphatase activity; SA, represents invertase activity, UA, represents urease activity; CA, represents catalase activity; SOB, represents Sobs index; SHA, represents Shannon index; SIM represents Simpson index; ACE, represents Ace index; CHA, represents Chao index; COV, represents Coverage index.

The interaction between yield and soil indexes was analyzed by Correlation analysis method. The correlation between each index was visually presented by color and significance symbol, *P* < 0.05 significant, *P* < 0.01 extremely significant, and *P* < 0.001 extremely significant, as shown in **Figure 13**.

It can be concluded from **Figure 13** that the grain yield was significantly positively correlated with catalase activity, and the correlation coefficient was 0.85. It was significantly positively correlated with biomass, organic matter content, alkaline phosphatase activity and sucrase activity, and the correlation coefficients were 0.73, 0.73, 0.78 and 0.73, respectively. It was significantly positively correlated with soil moisture content and urease activity, and the correlation coefficients were 0.59 and 0.60, respectively. It was significantly negatively correlated with pH value, and the correlation coefficient was -0.74; there was a significant negative correlation with conductivity and water soluble salt, and the correlation coefficients were -0.69 and -0.66, respectively. There was a significant positive correlation between biomass and catalase activity, and the correlation coefficient was 0.85. It was significantly positively correlated with urease activity and sucrase activity, and the correlation coefficients were 0.73 and 0.60, respectively. There was a significant negative correlation with conductivity, and the correlation coefficient was -0.69.

The soil pH, electrical conductivity and water soluble salt decrease and the soil organic matter and soil enzyme activity increase, improve soil structure, and increase yield by co-inoculation of *Bacillus subtilis* and *Rhizobium*. It is effective measures to improve catalase activity, organic matter content, alkaline phosphatase activity, sucrase activity and urease activity, reduce pH value, electrical conductivity and water soluble salt by analyzing the interaction between soil indicators and yield for achieving high yield and improving soil ecological environment.

## 4 Discussion

### 4.1 Co-inoculation increased yield and dry matter accumulation

The results in paper showed that the yield and dry matter accumulation of soybean in T3 treatment were the highest, with values of 3182.67 kg/hm^2^ and 19622.75 kg/hm^2^(**Table 2**, **Figure 3**), respectively. This data fully shows that the treatment of *Bacillus subtilis* and *Rhizobium* co-inoculation combined with fertilization has a significant effect on promoting the accumulation of dry matter in soybean. *Bacillus subtilis* 8–32 strains secrete IAA and siderophores, which have a good effect on fungal diseases and enhance nutrient absorption (Sapna et al., 2023). *Rhizobium* enhances host salt tolerance through nodulation signaling molecules and provides ammonium nitrogen to support growth (Wang et al., 2022). Co-inoculation of *B. subtilis* and *Rhizobium* can promote plant growth and significantly alleviate the negative effects of salt stress on soybean. The synergistic mechanisms include: *B. subtilis* can produce IAA and siderophores, improve plant nutritional status, and reduce plant diseases under stress conditions; *Rhizobium* directly provides nitrogen source through symbiotic nitrogen fixation; the co-inoculation of *Bacillus subtilis* and *Rhizobium* can improve the rhizosphere microbial community structure, enhance the health and function of plant roots, promote plant absorption of nutrients, enhance metabolic function, and improve plant tolerance to salt stress (eMaymon et al., 2015).

Co-inoculation of *Bacillus subtilis* and *Rhizobium* can improve soil properties, promote plant growth, increase nutrient cycling, and affects plant biomass (Jia et al., 2025). Chen et al (Chen et al. (2025) showed that co-inoculation of *Bacillus subtilis* and *Rhizobium* could increase crop yield, and the fresh weight of Chinese cabbage treated with bacterial agent was significantly increased by 14.32% compared with the control group. Zhang et al. (Zhang C, et al., 2024) showed that *Bacillus subtilis* or *Rhizobium*, applied alone or together, significantly increased chili pepper yield, with co - inoculation raising it by 29.89% over the control. Peng et al. (2025) showed that the co-inoculation of *Bacillus subtilis* and *Rhizobium* significantly increased the aboveground biomass of cucumber plants, compared with the control group, fresh weight and dry weight increased by 3–4 times. The cucumber yield in the second and third growing season of the treatment (T4) co-inoculated *Bacillus subtilis* and *Rhizobium* was 17.43% and 14.63% higher than that of the treatment (VM) only with organic fertilizer. Zhao et al. (2025) showed that co-inoculation of *Bacillus subtilis* and *Rhizobium* increased seed cotton yield by 9.1% in mild salt stress. It has been proved to be effective in alleviating saline-alkali stress and promoting soybean yield and dry matter accumulation by improving plant physiological metabolism and soil microenvironment to apply co-inoculation of *Bacillus subtilis* and *Rhizobium* (Sarwar et al., 2025). Fernando et al. (Fernando et al., 2021) found that bioinoculants modify soybean root architecture, enhance osmotic regulation and boost dry matter accumulation and yield in salt stress. Fernando et al. (2023) showed that the yield of soybean co-inoculated with bacterial agents was significantly higher than that of the control group, which was 31% higher than that of the control group. The yield increase effect of co-inoculation was better than that of single inoculation of *Rhizobium* or nitrogen-fixing bacteria. Lamptey et al. (2014) showed that the grain yield of soybean was significantly increased by the application of *Rhizobium*, and the yield was increased to 1262 kg/ha after the application of *Rhizobium*, compared with the treatment without *Rhizobium*, the increase was about 21%.

In the process of soybean growth, the number of pods per plant, the number of grains per pod and the weight of 100 grains are the key indicators to measure the yield. By co-inoculating and using two kinds of *Bacillus subtilis* and *Rhizobium*, it can not only effectively increase the nutrient supply of soybean plants, but also significantly promote the development of their roots and enhance the activity of enzymes in the soil. These combined effects ultimately promoted the full growth of pods and the accumulation of dry matter in grains, thereby significantly increasing the overall yield of soybean. The research results provide a new technical idea for soybean planting and have important practical guiding significance.

### 4.2 Effects of co-inoculation on physicochemical properties of soybean rhizosphere soil in coastal saline-alkali land

Salinization can cause osmotic stress and oxidative damage, affect seed germination, growth and organ development of crops, and even lead to crop death (Zhang et al., 2024). The global saline-alkali land area is large and growing, which seriously threatens for food and environmental security (Bi et al., 2025). Co-inoculation of *Bacillus subtilis* and *Rhizobium* as a green improvement method can achieve soil remediation and promote crop growth by regulating the rhizosphere microenvironment (Zhou et al., 2024). Raji and Thangavelu (Raji and Thangavelu, 2022) found that double inoculation could significantly reduce soil pH and increase soil organic matter content. Wu et al. (2021) showed that strain Bac-9 formed a biofilm through EPS, converting soluble salts into insoluble complexes, and the water soluble salt was reduced by 19.7%. *Rhizobium* promoted the vertical migration of salt by enhancing root activity and improving soil permeability. Omara et al. (2022) found that the water soluble salt of the compound microbial agent treatment group was 12%-18% lower than that of the single microbial agent in the saline-alkali soil wheat experiment. The results in paper showed that the application of *Bacillus subtilis* and *Rhizobium* co-inoculation treatment significantly reduced the soil pH value, electrical conductivity and water soluble salt, and significantly increased the soil organic matter content at the maturity stage of soybean, among which T3 treatment had the best effect (**Table 3**). This trend is consistent with the results of previous studies, which effectively improves saline-alkali land and improves soil quality.

In this study, Jidou 12 with strong saline-alkali tolerance was used to conduct a field experiment in Lizizha Village of coastal saline-alkali land to explore the effects of co-inoculation of two *Bacillus subtilis* and *Rhizobium* on soil physical and chemical properties. It was found that these two microbial agents effectively improved the rhizosphere environment of soybean in saline-alkali land by acidification of rhizosphere, fixation of salt ions and increase of organic matter input. *Bacillus subtilis* mainly affects water transport by directly improving soil structure. The organic acids secreted by *Bacillus subtilis* can neutralize soil alkaline ions, reduce pH, and replace Na in soil colloids to reduce the contribution of sodium ions to soil alkalinity. *Rhizobium* nitrogen fixation provides nitrogen, promotes soybean growth, increases transpiration to dilute rhizosphere salt concentration, and forms a biofilm when symbiosis with the host to isolate the direct stress of salt on the root system. At the same time, *Rhizobium* increased soybean biomass through symbiotic nitrogen fixation, increased organic matter after plant residues returned to soil, and the photosynthetic products provided by host plants to *Rhizobium* entered the soil through root exudates, further increasing soil organic matter (Maymon et al., 2015). In this study, the two microbial agents had a significant effect on improving soil organic matter, which may be related to the large number of leaves falling off at the mature stage of soybean. Co-inoculation of *Bacillus subtilis* and *Rhizobium* effectively improved soil environment, increased soil nutrient availability, and enhanced plant stress resistance, thereby reducing the adverse effects of saline-alkali stress on soybean growth and yield.

### 4.3 Effects of co-inoculation on enzyme activities in rhizosphere soil of soybean in coastal saline-alkali land

Saline-alkali stress inhibits urease, dehydrogenase and alkaline phosphatase activities and hinders soil nitrogen and phosphorus cycling (Li et al., 2019). The co-inoculation of *Bacillus subtilis* and *Rhizobium* alleviated the negative effects of salt stress on crops to a certain extent (Kumar et al., 2024), and regulated the rhizosphere microecology by increasing soil enzyme activity. *Bacillus subtilis* promotes the dissolution of insoluble phosphorus in soil by secreting organic acids and phosphatases, and significantly increases alkaline phosphatase activity (Liu et al., 2022). Hussain et al. (2023) showed that the enzyme activity (such as SOD and CAT) of plants increased significantly and the level of oxidative stress markers was reduced enhancing the salt stress tolerance of plants by the co-inoculation of both microbial agents. Xu et al. (2022) showed that the activities of catalase, superoxide dismutase and peroxidase could significantly increase which enhance the salt tolerance and growth ability of plants by the co-inoculation of bacterial agents. Salt-tolerant Streptomyces D2-8 (similar to *Rhizobium* function) dissociated from the rhizosphere of Phragmites australis by Guo et al. (2022) can significantly increase catalase activity in soybean rhizosphere and reduce oxidative damage caused by saline-alkali stress. Yan et al. (2025) showed that the sucrase activity of vineyard in saline-alkali stress significantly increased by co-inoculation of *Bacillus subtilis* and *Rhizobium*. The soil sucrase activity of the co-inoculation treatment group was significantly higher than that of the control group by 4.12%. Gao et al. (2023) showed that the activities of superoxide dismutase (SOD), peroxidase (POD) and catalase (CAT) significantly increased by co-inoculation.

In this study, through field experiments, it was found that single application of *Bacillus subtilis* or *Rhizobium* could increase soil alkaline phosphatase activity, among which *Bacillus subtilis* had a better effect, while the effect of co-inoculation was the most significant, with a value of 12.22 mg/(g·d) (**Figure 4**). In terms of sucrose enzyme activity, single application of *Bacillus subtilis* had a promoting effect, and the effect was more obvious when co-inoculated, with a value of 12.11 mg/(g·d) (**Figure 4**). For urease activity, single application of *Rhizobium* could increase its activity, and the effect of co-inoculation was more significant, with a value of 0.61 mg/(g·d) (**Figure 4**). Moreover, biological inoculants significantly increased catalase activity. In general, the combination of *Bacillus subtilis* and *Rhizobium* achieved better results through functional complementation, and the soil enzyme activity of T3 treatment was the highest. The alkaline phosphatase, urease, and catalase activities of T3 treatment were significantly higher than that of the control. Its sucrose enzyme activity was also significantly higher than that of T2 and the control. Considering that the high saline-alkali environment of coastal saline-alkali land may inhibit the nodulation ability of nodules, thereby reducing the efficiency of nitrogen fixation and indirectly affecting the induction of sucrase by root exudates, T3 treatment has excellent performance in improving soil enzyme activity. Co-inoculation helps to alleviate salt stress, promote nutrient cycling, improve soil fertility and structure, create a better soil environment for soybean growth, and ultimately achieve the goal of increasing yield.

### 4.4 Effects of co-inoculation on bacterial community in rhizosphere soil of soybean in coastal saline-alkali land

In this study, co-inoculation with T3 treatment significantly improved the α diversity of rhizosphere soil bacterial communities (**Table 4**). Soybean rhizosphere bacterial community constitutes the core of the interaction between soybean and soil. A healthy microbial community structure can promote the growth and development of roots, improve the absorption efficiency of nutrients and water, inhibit the activity of pathogens, reduce the risk of disease, and thus increase the yield of soybean (Moretti et al., 2024). The relative abundance of Pseudomonadota in T1 treatment was significantly higher than that in other treatments. For Actinomycetota, the relative abundance of T2 treatment was significantly higher than that of T1 treatment, and the relative abundance of Actinomycetota in each treatment showed no significant difference. In addition, the relative abundance of Bacillota in T2 treatment was significantly higher than that in T1 treatment (**Figure 6**). Pseudomonadota, Actinomycetota, and Bacillota rely on functional synergy, hierarchical regulation and stress adaptability to build a core microbial network that supports high yield of soybeans, and achieve the green production goal of ‘adjusting soil with bacteria and increasing yield with bacteria’ (da Silva et al., 2025). In T1 treatment, the relative abundance of Ensifer was significantly higher than that of other treatment groups. In T3 treatment, the relative abundance of Sphingomonas was significantly higher than that of T1 and T2 treatments. In addition, the relative abundance of Bacillus in T2 treatment was significantly higher than that in other treatments (**Figure 7**). Ensifer supports plant growth through nitrogen fixation, while Sphingomonas degrades toxins to maintain nitrogen fixation efficiency. Under saline-alkali stress, Bacillus can reduce ethylene toxicity, Sphingomonas can stabilize cell membrane, and Ensifer can maintain nitrogen fixation activity. These three genera provide a microbial support system for high yield and high efficiency of soybean through the synergistic effect of nitrogen fixation and energy supply, detoxification and growth promotion, disease resistance and stable yield (Oliveira et al., 2025).

Soil bacterial community is an important part of farmland soil ecosystem, and its community structure and functional diversity are closely related to soil environmental quality (Qin et al., 2024). Salinity is a key factor affecting the microbial community. It not only directly affects the growth and metabolism of microorganisms and changes the osmotic pressure of cells, but also indirectly affects the community structure by regulating the physical and chemical properties of soil (Li et al., 2024). Co-inoculation of *Bacillus subtilis* and *Rhizobium* can effectively improve soil microbial diversity and fertility (Khan et al., 2024). As an important biological strategy for the improvement of saline-alkali land, *Bacillus subtilis* and *Rhizobium* have a positive effect on the regulation of bacterial community in soybean rhizosphere soil. Fernando et al. (2021) found that root architecture of soybean (such as increased lateral roots) could change by inoculation with *Bacillus subtilis*, expand the microbial niche and promote the enrichment of phosphate-solubilizing bacteria and actinomycetes, which may be achieved through the supply of carbon sources for root exudates (such as sugars and organic acids), and the preferential proliferation of bacteria to Firmicutes in salt stress. *Rhizobium* forms a mutually beneficial relationship with the host through symbiotic nitrogen fixation, and signal molecules such as flavonoids released during the nodulation process can specifically recruit functional bacteria to form a mutually beneficial network (Suárez et al., 2024). Raji and Thangavelu (Raji and Thangavelu, 2022) found that double inoculation also significantly increased the abundance of potassium-solubilizing microorganisms in soil, alleviated potassium limitation in salt stress, and indirectly improved soil structure.

The stability of the combined application of bacteria is restricted by environmental factors (Chand et al., 2022). When the salt concentration was more than 5 dS/m, the microbial colonization was inhibited. When the salt concentration was more than 9 dS/m, the effect was significant. When the salt concentration was 29.9 dS/m, the microbial residue was almost reduced by more than 90% (Shao et al., 2022). *Bacillus subtilis* and *Rhizobium* bacteria regulate the rhizosphere microbial community of soybean in saline-alkali land through differential pathways, and the combined application shows synergistic potential. However, environmental dependence and mechanism complexity are still the bottleneck of practical application (Mažylytė et al., 2024). The abundance of bacterial communities of T3 treatment in soybean rhizosphere soil is general, which are somewhat different from the previous views and may be caused by the application of foreign strains affecting indigenous strains, complex field environment and uncontrollable factors. It forms an interaction relationship to combine inoculation of *Bacillus subtilis* and *Rhizobium*, which can secrete a variety of enzymes and antibiotics. These substances can not only inhibit the growth of harmful microorganisms, but also promote the proliferation of beneficial bacteria (Zhang et al., 2023), thereby improving the composition and function of the rhizosphere microbial community, creating a more favorable microbial environment for soybean growth, and having a positive effect on soybean yield.

The colonization efficiency of microbial communities is affected by foreign bacteria. In order to achieve efficient compatibility and stable colonization of inoculants and rhizosphere indigenous microbial communities, multi-dimensional coordinated control strategies need to be adopted. The primary approach is to select functional strains adapted to the local environment and construct a synthetic microbial community with a mutually beneficial symbiotic relationship, at the same time, the colonization resistance of indigenous communities was weakened by applying organic amendments to regulate the rhizosphere microenvironment. Finally, the precise regulation and ecological function enhancement of rhizosphere microbial community were realized.

### 4.5 The interaction relationship between soil index and yield

The growth of soybean could be seriously restricted because soil salt content is high and barren in coastal saline-alkali land, while the co-inoculation of *Bacillus subtilis* and *Rhizobium* can significantly promote the formation of soybean yield by improving the rhizosphere soil environment, regulating the microbial community and enhancing the plant resistance (Zhang et al., 2024). In agricultural production, co-inoculation of *Bacillus subtilis* and *Rhizobium* can effectively regulate soil properties and increase crop yield. Specifically, soil pH, electrical conductivity, and water soluble salt reduce and soil organic matter content, and soil enzyme activity, including catalase, alkaline phosphatase, sucrase, and urease increase by co-inoculation of *Bacillus subtilis* and *Rhizobium*, which help to improve soil structure and create more favorable conditions for crop growth.

Through the correlation analysis of soil indicators and yield, the interaction mechanism was explored (**Figure 13**). The contribution of co-inoculation of *Bacillus subtilis* and *Rhizobium* to the improvement of soybean yield in coastal saline-alkali land is essentially a multi-dimensional synergy of the’ soil-microorganism-plant’ system: co-inoculation of *Bacillus subtilis* and *Rhizobium* improves the soil environment so that root configuration optimization and nutrient absorption enhancement promote microbial-plant signal interaction(such as hormones, metabolites) to improve stress resistance and ultimately achieve the purpose of increasing yield. Co-inoculation of *Bacillus subtilis* and *Rhizobium* significantly improved the rhizosphere soil environment of soybean in coastal saline-alkali land through the chain reaction of ‘salt reduction-soil improvement-growth promotion-stress resistance’, and finally achieved yield increase by regulating microbial function and plant physiological metabolism (Sapna et al., 2023; Wang et al., 2022).

Single application or co-inoculation of two biological agents could significantly increase catalase activity. Increasing the application amount of organic fertilizer or straw returning could increase soil organic matter content. Co-inoculation of *Bacillus subtilis* and *Rhizobium* could significantly increase alkaline phosphatase activity. Single application of *Bacillus subtilis* or co-inoculation of two biological agents could significantly increase sucrase activity. Single application of *Rhizobium* or co-inoculation of two biological agents could significantly increase urease activity. The application of desulfurization gypsum can reduce soil pH, and the leaching of soil by natural rainfall can effectively reduce soil conductivity and total water-soluble salt (Lu et al., 2025). In summary, a series of scientific methods, such as planting soybean in coastal saline-alkali land, applying biological agents, rational fertilization (increasing the amount of organic fertilizer, appropriately applying chemical fertilizers containing nitrogen, phosphorus, potassium and other nutrient elements), straw returning, applying biochar, and applying desulfurization gypsum, can optimize farmland management and increase crop yield (Siddique et al., 2025).

## 5 Conclusion

The field experiment of the effects of co-inoculation of *Bacillus subtilis* and *Rhizobium* on soybean yield and soil bacterial community was carried out in Huanghua City, Hebei Province, which belongs to coastal saline-alkali land. The soybean yield and dry matter quality, physical and chemical properties and bacterial community of soybean rhizosphere soil were determined. The main conclusions are as follows by analyzing experimental data:

1. Variation regularity do yield and dry matter accumulation in co-inoculation. The application of *Bacillus subtilis* or *Rhizobium* can increase soybean yield. The co-inoculation of *Bacillus subtilis* and *Rhizobium* has a better effect on increasing yield than single application. The co-inoculation significantly increased soybean yield and dry matter accumulation. The values were 3182.67 kg/hm^2^ and 19622.75 kg/hm^2^, respectively, which were 18.03% and 8.93% higher than the control group.
2. Improvement enzyme activity and soil microbial communities in co-inoculation. Co-inoculation improved the physicochemical properties of saline - alkaline soil. Single inoculation with *Bacillus subtilis* or *Rhizobium* increased alkaline phosphatase activity. *Bacillus subtilis* also boosted sucrose enzyme activity, while *Rhizobium* enhanced urease activity. Single inoculation or co-inoculation significantly increased catalase activity, with co-inoculation being the most effective. Compared to the control, co-inoculation increased alkaline phosphatase, sucrose enzyme, urease, and catalase activities by 14.9%, 22.4%, 15.1%, and 5.2%, respectively. Furthermore, co-inoculation increased bacterial diversity and formed mutualistic networks.
3. Interaction between yield and soil indicators was quantified. Correlation analysis of the interaction between soil indicators and yields showed that grain yield was extremely positively correlated with catalase activity, biomass, organic matter, alkaline phosphatase activity, and sucrose enzyme activity. It was extremely negatively correlated with pH and significantly negatively correlated with electrical conductivity and water soluble salt. Crop yield could be increased and soil quality could be improved by increasing catalase activity and organic matter, alkaline phosphatase, sucrose enzyme, and urease activities, while reducing pH, electrical conductivity, and water soluble salt.

The promotion of salt-tolerant varieties (Jidou 12) combined with the co-inoculation mode of *Bacillus subtilis* and *Rhizobium* in coastal saline-alkali land can effectively achieve the goals of increasing yield, improving soil quality and improving saline-alkali land, which provides a new paradigm for the green production technology of soybean in saline-alkali land. In the future, attention should be paid to the regulation of rhizosphere microenvironment by adding organic modifiers, and the survival and competitiveness of microbial agents should be systematically improved by using microcapsule embedding and precise delivery technology, so as to finally realize the precise regulation of rhizosphere microbial community structure and the enhancement of ecological functions.

## Data Availability

The datasets presented in this study can be found in online repositories. The names of the repository/repositories and accession number(s) can be found in the article/supplementary material.

## References

[B1] Ali S. Khan M. Moon Y. S. (2025). Synergistic Effect of Serratia fonticola and Pseudomonas koreensis on Mitigating Salt Stress in Cucumis sativus L. Curr. Issues Mol. Biol. 47, 194–194. doi: 10.3390/cimb47030194, PMID: 40136448 PMC11941737

[B2] Bao S. (2000). Soil Agrochemical Analysis (Beijing: China Agricultural Publishing House).

[B3] Bi W. Sun Y. Yao Z. Zhao Z. Niu Y. (2025). Bacillus halophilus BH-8 combined with coal gangue as a composite microbial agent for the rehabilitation of saline-alkali land. Microorganisms 13, 532–532. doi: 10.3390/microorganisms13030532, PMID: 40142425 PMC11945998

[B4] Chand K. K. Inderjeet S. Sharon N. Poonam S. Kumar G. R. Asmita S. (2022). Co-inoculation of indigenous Pseudomonas oryzihabitans and Bradyrhizobium sp. modulates the growth, symbiotic efficacy, nutrient acquisition, and grain yield of soybean. Pedosphere 32, 438–451. doi: 10.1016/S1002-0160(21)60085-1

[B5] Chen Z. Zhang H. Lv W. Zhang S. Du L. Li S.. (2025). Bacillus velezensis SS-20 as a potential and efficient multifunctional agent in biocontrol, saline-alkaline tolerance, and plant-growth promotion. Appl. Soil Ecol. 205, 105772–105772. doi: 10.1016/j.apsoil.2024.105772

[B6] Cheng Y. Jiang X. He X. Wu Z. Lv Q. Zhao S.. (2025). Bacillus velezensis 20507 promotes symbiosis between Bradyrhizobium japonicum USDA110 and soybean by secreting flavonoids. Front. Microbiol. 16, 1572568. doi: 10.3389/fmicb.2025.1572568, PMID: 40212382 PMC11983421

[B7] da Silva M. S. R. A. de Carvalho L. A. L. Santos C. H. B. Frezarin E. T. da Silva C. G. N. Pinheiro D. G.. (2025). Effect of co-inoculation with plant growth-promoting bacteria on the microbiome of soybean roots. Front. Sustain. Food Syst. 9, 1505001. doi: 10.3389/fsufs.2025.1505001

[B8] Fernando A. F. Aurenivia B. Guimarães B. L. William M. L. Ferreira A. A. S. (2021). Bacillus subtilis changes the root architecture of soybean grown on nutrient-poor substrate. Rhizosphere 18. doi: 10.1016/J.RHISPH.2021.100348

[B9] Fernando M. B. Zampar E. J. D. O. de Almeida Junior J. H. V. Rahmen C. B. M. A. Takeyoshi I. T. Augusto B. M. (2023). Effect of different methods of inoculation and co-inoculation of;Bradyrhizobium;spp. and;Azospirillum brasilense;on soybean agronomic performance in fields with a history of inoculation. Arch. Agron. Soil Sci. 69, 2925–2937. doi: 10.1080/03650340.2023.2184807

[B10] Fonseca de Souza L. Oliveira H. G. Pellegrinetti T. A. Mendes L. W. Bonatelli M. L. Dumaresq A. S. R.. (2025). Co-inoculation with Bacillus thuringiensis RZ2MS9 and rhizobia improves the soybean development and modulates soil functional diversity. FEMS Microbiol. Ecol. 101, 1–12. doi: 10.1093/FEMSEC/FIAF013, PMID: 39844349 PMC11796456

[B11] Gao H. Yang D. Yang L. Han S. Liu G. Tang L.. (2023). Co-inoculation with Sinorhizobium meliloti and Enterobacter ludwigii improves the yield, nodulation, and quality of alfalfa (Medicago sativa L.) under saline-alkali environments. Ind. Crops Products 199. doi: 10.1016/J.INDCROP.2023.116818

[B12] Guo L. Nie Z. Zhou J. Zhang S. An F. Zhang L.. (2022). Effects of different organic amendments on soil improvement, bacterial composition, and functional diversity in saline-sodic soil. Agronomy-Basel 12, 2294. doi: 10.3390/agronomy12102294

[B13] Hu Z. Si W. (2025). International experience and enlightenment of sustainable development of soybean industry chain. World Agric. 01), 67–78. doi: 10.13856/j.cn11-1097/s.2025.01.006

[B14] Hussain S. I. Ali S. I. Asad R. Khalid H. M. Gadah A. Aamir M. M.. (2023). Co-application of copper oxide nanoparticles and Trichoderma harzianum with physiological, enzymatic and ultrastructural responses for the mitigation of salt stress. Chemosphere 336, 139230–139230. doi: 10.1016/j.chemosphere.2023.139230, PMID: 37343643

[B15] Jia Z. Li C. Zhang S. Tang Y. Ma S. Liu X.. (2025). Microbial inoculants modify the functions of soil microbes to optimize plant growth at abandoned mine sites. J. Environ. Sci. 154, 678–690. doi: 10.1016/j.jes.2024.10.002, PMID: 40049907

[B16] Khan A. Singh A. V. Kukreti B. Pandey D. T. Upadhayay V. K. Kumar R.. (2024). Deciphering the impact of cold-adapted bioinoculants on rhizosphere dynamics, biofortification, and yield of kidney bean across varied altitudinal zones. Sci. total Environ. 927, 172204–172204. doi: 10.1016/j.scitotenv.2024.172204, PMID: 38580128

[B17] Kumar A. Yadav A. Dhanda P. S. Delta A. K. Sharma M. Kaushik P. (2024). Salinity stress and the influence of bioinoculants on the morphological and biochemical characteristics of faba bean (Vicia faba L.). Sustainability 14, 14656.

[B18] Lamptey S. Ahiabor B. D. K. Yeboah S. Osei D. (2014). Effect of rhizobium inoculants and reproductive growth stages on shoot biomass and yield of soybean (Glycine max (L.) merril). J. Agric. Sci. 6, 44. doi: 10.5539/jas.v6n5p44

[B19] Li Z. He X. (2024). Study on the influencing factors and optimization countermeasures of the spatial and temporal evolution pattern of soybean production in China. Soybean Sci. 43, 782–792.

[B20] Li Q. Yang A. Zhang W.-H. (2019). Higher endogenous bioactive gibberellins and α-amylase activity confer greater tolerance of rice seed germination to saline-alkaline stress. Environ. Exp. Bot. 162, 357–363. doi: 10.1016/j.envexpbot.2019.03.015

[B21] Li Y. Zhang D. Wen Y. Liu X. Zhang Y. Wang G. (2024). Spatiotemporal patterns and driving factors of carbon footprint in coastal saline cropland ecosystems: A case study of the yellow river delta, China. Land 13, 2145–2145. doi: 10.3390/land13122145

[B22] Li M. Zhou W. Sun M. Shi W. Lun J. Zhou B.. (2024). Decoupling soil community structure, functional composition, and nitrogen metabolic activity driven by salinity in coastal wetlands. Soil Biol. Biochem. 198, 10954–10957. doi: 10.1016/j.soilbio.2024.109547

[B23] Liu Y. X. Jiang X. L. Xu Y. N. Piao X. C. Lian M. L. (2022). Antibacterial mechanisms of Orostachys cartilaginous cell cultures: effect on cell permeability and respiratory metabolism of Bacillus subtilis. Plant Cell Tissue Organ Culture (PCTOC) 148, 189–196. doi: 10.21203/rs.3.rs-226560/v1

[B24] Liu W. Wang Q. Hou J. Tu C. Luo Y. Peter C. (2016). Whole genome analysis of halotolerant and alkalotolerant plant growth-promoting rhizobacterium Klebsiella sp. D5A. Sci. Rep. 6, 26710. doi: 10.1038/srep26710, PMID: 27216548 PMC4877636

[B25] Lu P. Liu Y. Zhang Z. Wang C. Huang M. Xia C. (2025). Subsurface pipe drainage and straw mulching synergistically enhance greenhouse-scale soil leaching via seasonal rainfall. Irrigation Sci., 1–24. doi: 10.1007/s00271-025-01032-x

[B26] Ma H. Liu X. Zhang R. Li M. Li Q. Ding X.. (2025). Function of nodulation-associated gmNARK kinase in soybean alkali tolerance. Int. J. Mol. Sci. 26, 325–325. doi: 10.3390/ijms26010325, PMID: 39796181 PMC11719578

[B27] Maymon M. Martínez-Hidalgo P. Tran S. S. Ice T. Craemer K. Anbarchian T.. (2015). Mining the phytomicrobiome to understand how bacterial coinoculations enhance plant growth. Front. Plant Sci. 6, 784. doi: 10.3389/fpls.2015.00784, PMID: 26442090 PMC4585168

[B28] Mažylytė R. Kailiuvienė J. Mažonienė E. Orola L. Kaziūnienė J. Mažylytė K.. (2024). The co-inoculation effect on triticum aestivum growth with synthetic microbial communities (SynComs) and their potential in agrobiotechnology. Plants (Basel Switzerland) 13, 1716–1716. doi: 10.3390/plants13121716, PMID: 38931148 PMC11207813

[B29] Moretti L. G. Crusciol C. A. C. Leite M. F. A. Momesso L. Bossolani J. W. Costa O. Y. A.. (2024). Diverse bacterial consortia: key drivers of rhizosoil fertility modulating microbiome functions, plant physiology, nutrition, and soybean grain yield. Environ. Microbiome 19, 50–50. doi: 10.1186/s40793-024-00595-0, PMID: 39030648 PMC11264919

[B30] Nader A. A. Hauka F. I. A. Afify A. H. Sawah A. M. E. (2024). Drought-Tolerant Bacteria and Arbuscular Mycorrhizal Fungi Mitigate the Detrimental Effects of Drought Stress Induced by Withholding Irrigation at Critical Growth Stages of Soybean (Glycine max, L.). Microorganisms 12, 1123–1123. doi: 10.3390/microorganisms12061123, PMID: 38930505 PMC11205826

[B31] Oliveira C. E. D. S. Jalal A. Boleta G. H. M. Ito W. C. N. Fernandes G. C. da Silva M. V.. (2025). Rhizobacteria co-inoculation methods improve grain yield, nutrient absorption and leaf gas exchange in soybean. Int. J. Plant Production 19, 1–22.

[B32] Omara A. E.-D. Hafez E. M. Osman H. S. Rashwan E. El-Said M. A. A. Alharbi K.. (2022). Collaborative impact of compost and beneficial rhizobacteria on soil properties, physiological attributes, and productivity of wheat subjected to deficit irrigation in salt affected soil. Plants-Basel 11, 877–877. doi: 10.3390/plants11070877, PMID: 35406858 PMC9002696

[B33] Peng Y. Zhang H. Li G. Zhang J. (2025). Microbial inoculum improved soil aggregate formation and increased cucumber yield in a greenhouse under secondary salinization conditions. J. Environ. Manage. 376, 124576. doi: 10.1016/j.jenvman.2025.124576, PMID: 39970667

[B34] Petereit J. Bayer P. E. Tay Fernandez C. G. Batley J. Edwards D. (2025). Changes of gene content in four crop species during domestication and breeding. Agric. Commun. 3, 100077. doi: 10.1016/j.agrcom.2025.100077

[B35] Qin L. Ni B. Zou Y. Freeman C. Peng X. Yang L.. (2024). Deciphering soil environmental regulation on reassembly of the soil bacterial community during wetland restoration. Sci. Total Environ. 954, 176586–176586. doi: 10.1016/j.scitotenv.2024.176586, PMID: 39349191

[B36] Raiesi F. Beheshti A. (2014). Soil specific enzyme activity shows more clearly soil responses to paddy rice cultivation than absolute enzyme activity in primary forests of northwest Iran. Appl. Soil Ecol. 75, 63–70. doi: 10.1016/j.apsoil.2013.10.012

[B37] Raji M. Thangavelu M. (2022). Co-inoculation of halotolerant potassium solubilizing Bacillus licheniformis and Aspergillus violaceofuscus improves tomato growth and potassium uptake in different soil types under salinity. Chemosphere 294, 133718–133718. doi: 10.1016/j.chemosphere.2022.133718, PMID: 35077735

[B38] Sapna C. Sahabram D. Manik C. S. Dhiraj P. Raman P. Muthukaruppan G.. (2023). Complete genome sequencing of Bacillus subtilis (CWTS 5), a siderophore-producing bacterium triggers antagonistic potential against Ralstonia solanacearum. J. Appl. Microbiol. 134, 1–14., PMID: 37002541 10.1093/jambio/lxad066

[B39] Sarwar G. Fatima M. Danish S. Alharbi S. A. Ansari M. J. Alarfaj A. A. (2025). Enhancing wheat growth under chromium toxicity using gibberellic acid and microbial inoculants as modulating agents. Sci. Rep. 15, 8356–8356. doi: 10.1038/s41598-025-92828-6, PMID: 40069275 PMC11897154

[B40] Shao P. Han H. Sun J. Yang H. Xie H. (2022). Salinity effects on microbial derived-C of coastal wetland soils in the yellow river delta. Front. Ecol. Evol. 10, 872816. doi: 10.3389/FEVO.2022.872816

[B41] Shi X. Yan L. Yang C. Yan W. Moseley D. Wang T.. (2018). Identification of a major quantitative trait locus underlying salt tolerance in ‘Jidou 12’ soybean cultivar. BMC Res. Notes 11, 95. doi: 10.1186/s13104-018-3202-3, PMID: 29402302 PMC5800283

[B42] Siddique M. N. E. A. de Bruyn L. A. L. Osanai Y. Guppy C. N. (2025). Based on soil carbon saturation capacity what is the potential for soil carbon improvement in rice-based cropping systems of northwest region of Bangladesh. Geoderma Regional 42, 00975–00975. doi: 10.1016/j.geodrs.2025.e00975

[B43] Suárez M. M. Akyol T. Y. Nadzieja M. Andersen S. U. (2024). Increased diversity of beneficial rhizobia enhances faba bean growth. Nat. Commun. 15, 10673–10673. doi: 10.1038/s41467-024-54940-5, PMID: 39668214 PMC11638261

[B44] Wang X. Chen K. Zhou M. Gao Y. Huang H. Liu C.. (2022). GmNAC181 promotes symbiotic nodulation and salt tolerance of nodulation by directly regulating GmNINa expression in soybean. New Phytol. 236, 656–670. doi: 10.1111/nph.18343, PMID: 35751548

[B45] Wu L. Wang Y. Zhang S. Wei W. Kuzyakov Y. Ding X. (2021). Fertilization effects on microbial community composition and aggregate formation in saline-alkaline soil. Plant Soil 463, 523–535. doi: 10.1007/s11104-021-04909-w

[B46] Wu J. Wei Z. Zhao W. Zhang Z. Chen D. Zhang H.. (2023). Transcriptome analysis of the salt-treated actinidia deliciosa (A. Chev.) C. F. Liang and A. R. Ferguson plantlets. Curr. Issues Mol. Biol. 45, 3772–3786. doi: 10.3390/cimb45050243, PMID: 37232712 PMC10217562

[B47] Xu Y. Li Y. Long C. Han L. (2022). Alleviation of salt stress and promotion of growth in peanut by Tsukamurella tyrosinosolvens and Burkholderia pyrrocinia. Biologia 77, 2423–2433. doi: 10.1007/s11756-022-01073-z

[B48] Yan H. K. Zhang C. C. Nai G. J. Ma L. Lai Y. Pu Z. H.. (2025). Microbial inoculant GB03 increased the yield and quality of grape fruit under salt-alkali stress by changing rhizosphere microbial communities. Foods 14, 711–711. doi: 10.3390/foods14050711, PMID: 40077414 PMC11899072

[B49] Zhang J. Cai J. Xu D. Wu B. Chang H. Zhang B.. (2024). Soil salinization poses greater effects than soil moisture on field crop growth and yield in arid farming areas with intense irrigation. J. Cleaner Production 451, 142007. doi: 10.1016/j.jclepro.2024.142007

[B50] Zhang W. Mao G. Zhuang J. Yang H. (2023). The co-inoculation of Pseudomonas chlororaphis H1 and Bacillus altitudinis Y1 promoted soybean [Glycine max (L.) Merrill] growth and increased the relative abundance of beneficial microorganisms in rhizosphere and root. Front. Microbiol. 13, 1079348. doi: 10.3389/fmicb.2022.1079348, PMID: 36699592 PMC9868396

[B51] Zhang S. Shi B. (2024). The asymmetric tail risk spillover from the international soybean market to China’s soybean industry chain. Agriculture 14, 1198–1198. doi: 10.3390/agriculture14071198

[B52] Zhang C. Zhang L. Cao Y. Zhang S. Hou C. Zhang C. (2024). Effects of microbial organic fertilizer, microbial inoculant, and quicklime on soil microbial community composition in pepper (Capsicum annuum L.) continuous cropping system. Horticulturae 10, 1142–1142. doi: 10.3390/horticulturae10111142

[B53] Zhao X. Guo P. Wu X. Zhu M. Kang S. Du T.. (2025). Composite microbial agent improves cotton yield and resource use efficiency under mild salt stress by optimizing plant resource allocation. Agric. Water Manage. 310, 109358–109358. doi: 10.1016/j.agwat.2025.109358

[B54] Zhou Y. Li L. Wang J. Qi X. Fang H. Bai Y.. (2024). Effects of different microbial agent applications on the growth and quality of saffron (Crocus sativus L.) cormels. Scientia Hortic. 336, 113385. doi: 10.1016/j.scienta.2024.113385

[B55] Zhuang X. Liu Y. Fang N. Bai Z. Gao J. (2023). Quorum sensing improves the plant growth-promoting ability of Stenotrophomonas rhizophila under saline-alkaline stress by enhancing its environmental adaptability. Front. Microbiol. 14, 1155081. doi: 10.3389/fmicb.2023.1155081, PMID: 37113227 PMC10126360

